# Supplementary material for: Transparent integrated pyroelectric-photovoltaic structure for photo-thermo hybrid power generation
Source: Nat Commun. 2024 Apr 24;15:3466. doi: 10.1038/s41467-024-47483-2 (PMC11519937; doi:10.1038/s41467-024-47483-2)
Supplement: Supplementary file 1 — Supplementary Information [file 41467_2024_47483_MOESM1_ESM.pdf]

Supplementary Information:

Transparent integrated pyroelectric-photovoltaic structure for photo-thermo hybrid power generation

Malkeshkumar Patel,[1,2] Hyeong-Ho Park,[3] Priyanka Bhatnagar,[1,2] Naveen Kumar,[1,2] Junsik Lee,[1,2] and Joondong Kim[1,2,*]

[1] Photoelectric and Energy Device Application Lab (PEDAL), Multidisciplinary Core Institute for Future Energies (MCIFE), 119 Academy Rd. Yeonsu, Incheon 22012, Korea (Rep.)

[2] Department of Electrical Engineering, Incheon National University, 119 Academy Rd., Yeonsu, Incheon 22012, Korea (Rep.)

[3] Nanodevices Lab., Device Technology Division, Korea Advanced Nanofab Center (KANC), 109 Gwanggyo-ro, Yeongtong-gu, Suwon 16229, Korea (Rep.)

* E-mail: joonkim@incheon.ac.kr

Supplementary Table 1 | Summary of the elemental distribution of the transparent pyroelectric heterojunction device (TPHD) with AgNW/ZnO top electrode. This distribution is shown up to 305 nm in depth.

Distance (nm)	C	O	F	Si	Ag	Sn	Ni	Zn	Total
1.4081	89.8	7	0	2.99	0	0.21	0	0	100
7.0406	92.34	5	0	1.86	0.31	0.5	0	0	100
12.673	88.42	10.28	0	1.13	0.02	0.15	0	0	100
18.306	94.2	4.36	0	0.39	1.02	0.03	0	0	100
23.938	98.42	0	0.52	0.06	0.8	0.18	0	0.03	100
29.571	92.24	3.03	0.28	0	3.55	0.9	0	0	100
35.203	71.65	5	0.1	0	22.68	0	0	0.57	100
40.836	61.27	0	0	0.25	36.95	1.53	0	0	100
46.468	49.14	10.4	0.72	0.78	38.16	0.79	0	0	100
52.101	45.99	7.08	0	0.13	46.06	0.74	0	0	100
57.733	57.59	0	0	0.64	41.77	0	0	0	100
63.366	72.27	4.16	0.77	0	22.43	0.32	0	0.06	100
68.998	86.1	0.71	0.73	0.62	11.72	0.12	0	0	100
74.631	93.67	4.97	0	0	1.12	0.14	0	0.1	100
80.263	98.53	0.61	0.49	0.05	0	0.32	0	0	100
85.896	92.94	5.16	0.29	0.48	0.78	0.36	0	0	100
91.528	91.5	7	0	0	0.12	1.27	0	0.1	100
97.161	92.15	6.39	0	0.63	0.02	0.14	0	0.67	100
102.79	91.86	7.02	0	0	0.79	0.33	0	0	100
108.43	44.25	30.79	2.85	0	9.48	0.47	12.15	0	100
114.06	11.81	43.11	4.5	0.24	9.77	1.03	29.48	0.06	100
119.69	19.87	47.16	0	0	11.4	0.92	17.12	3.54	100
125.32	8.76	54	0.56	0.23	7.92	0.62	4.44	23.46	100
130.96	0.63	52.92	0	0	2.64	1.32	0	42.49	100
136.59	0	54.78	0	0	1.11	0	0	44.11	100
142.22	5.43	56.42	0	0	0.25	0	0	37.9	100
147.85	10.41	55.56	0.89	0.57	0.7	0.45	0	31.42	100
153.49	0	52.08	4.76	0.84	1.12	0.33	0	40.88	100
159.12	5.04	45.6	1.55	0	0.48	1.73	0	45.6	100
164.75	4.45	58.81	0	0	0.21	0	0	36.53	100
170.38	9.12	54.34	0	0.22	0.37	0.43	0	35.53	100
176.02	8.87	55.12	0	0.14	0.41	0.52	0	34.93	100
181.65	7.33	56.33	0	0.22	0.26	0.61	0	35.25	100
187.28	9.96	50.97	1.76	0	0.16	0.59	0	36.56	100

192.91	19.55	49.18	0	0	0.6	0	0	30.67	100
198.55	0.46	60.97	0	1.55	1.02	0.31	0	35.69	100
204.18	0.15	62.51	0	0.04	1.01	0.1	0	36.19	100
209.81	3.81	52.66	0	0.86	0.48	0	0	42.18	100
215.44	2.55	54.1	0	0	0	0.59	0	42.75	100
221.08	9.22	49.52	1.85	0.79	0	11.73	0	26.88	100
226.71	0	66.68	0	0.09	0.03	29.56	0	3.64	100
232.34	4.21	63.86	5.09	1.95	0.1	24.78	0	0	100
237.97	0	65.66	0	0	1.62	32.71	0	0	100
243.61	0	61.7	0	0	0.13	38.17	0	0	100
249.24	0	67.35	0	0	0	32.65	0	0	100
254.87	0	66.1	3.78	0.07	0	30.05	0	0	100
260.5	0	63.25	0	0	0.1	36.65	0	0	100
266.14	0	64.23	0	0.22	2.4	31.46	0	1.69	100
271.77	1.11	65.68	0	1.61	0.5	31.1	0	0	100
277.4	0	66.08	0	1.39	0	32.53	0	0	100
283.03	0	66.38	0	0	0.74	32.65	0	0.23	100
288.67	0	59.14	0	0	2.28	37.43	0	1.16	100
294.3	0	67.7	0	0	1.02	30.23	0	1.05	100
299.93	0	60.14	0	2.73	0.27	36.86	0	0	100
305.56	0	57.93	2.68	1.05	0	38.34	0	0	100

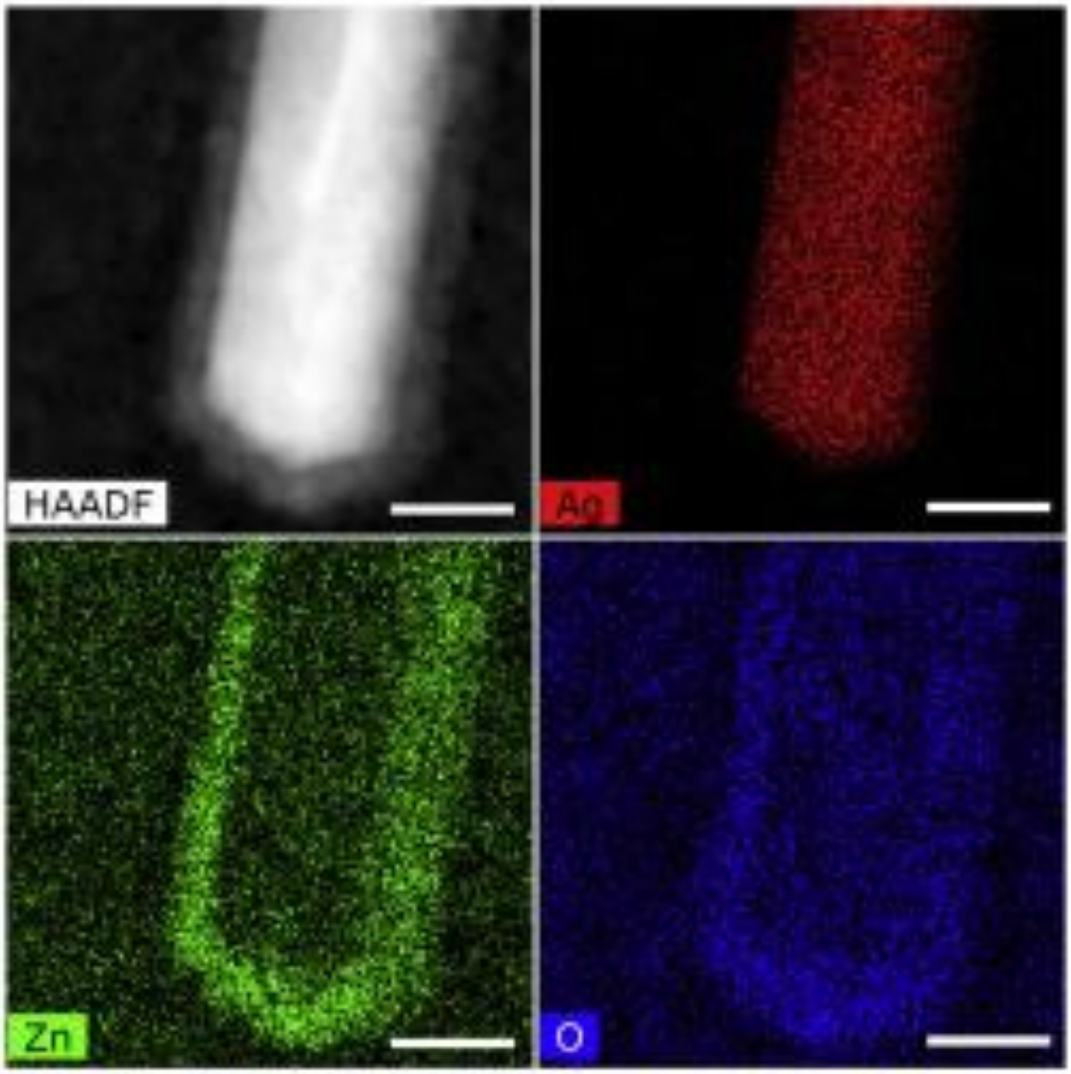

Supplementary Fig. 1 | High-angle annular dark-field (HAADF) image of AgNW/ZnO and elemental mapping of Ag, Zn, and O (scale bar, 30 nm).

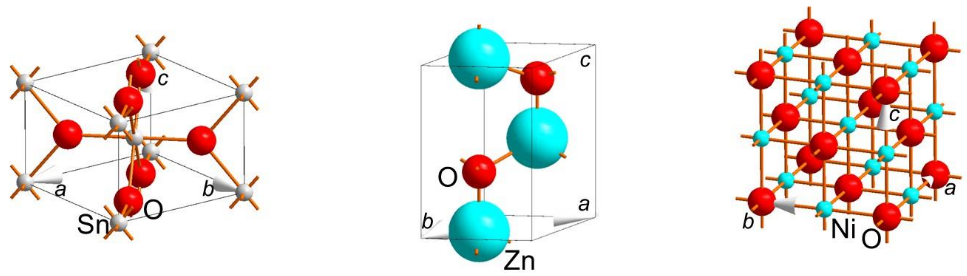

Formula sum	SnO_2	ZnO	NiO
Crystal system	Tetragonal	hexagonal	cubic
Space-group	P 42/m n m (136)	P 63 m c (186)	F m -3 m (225)
Cell parameters	a=4.7357 Å<br>c=3.1873 Å	a=3.2490 Å<br>c=5.2070 Å	a=4.18(1) Å
Cell ratio	a/b=1.00<br>b/c=1.4858<br>c/a=0.6730	a/b=1.00<br>b/c=0.6240<br>c/a=1.6026	a/b=1.00<br>b/c=1.00<br>c/a=1.00
Cell volume	71.48 Å^3	47.60 Å^3	73.03 Å^3

Supplementary Fig. 2 | Crystal structure and lattice parameters of SnO_2, ZnO, and NiO. These parameters were obtained from the COD database code 2104743 for SnO_2, 9011662 for ZnO, and 1010095 for NiO.

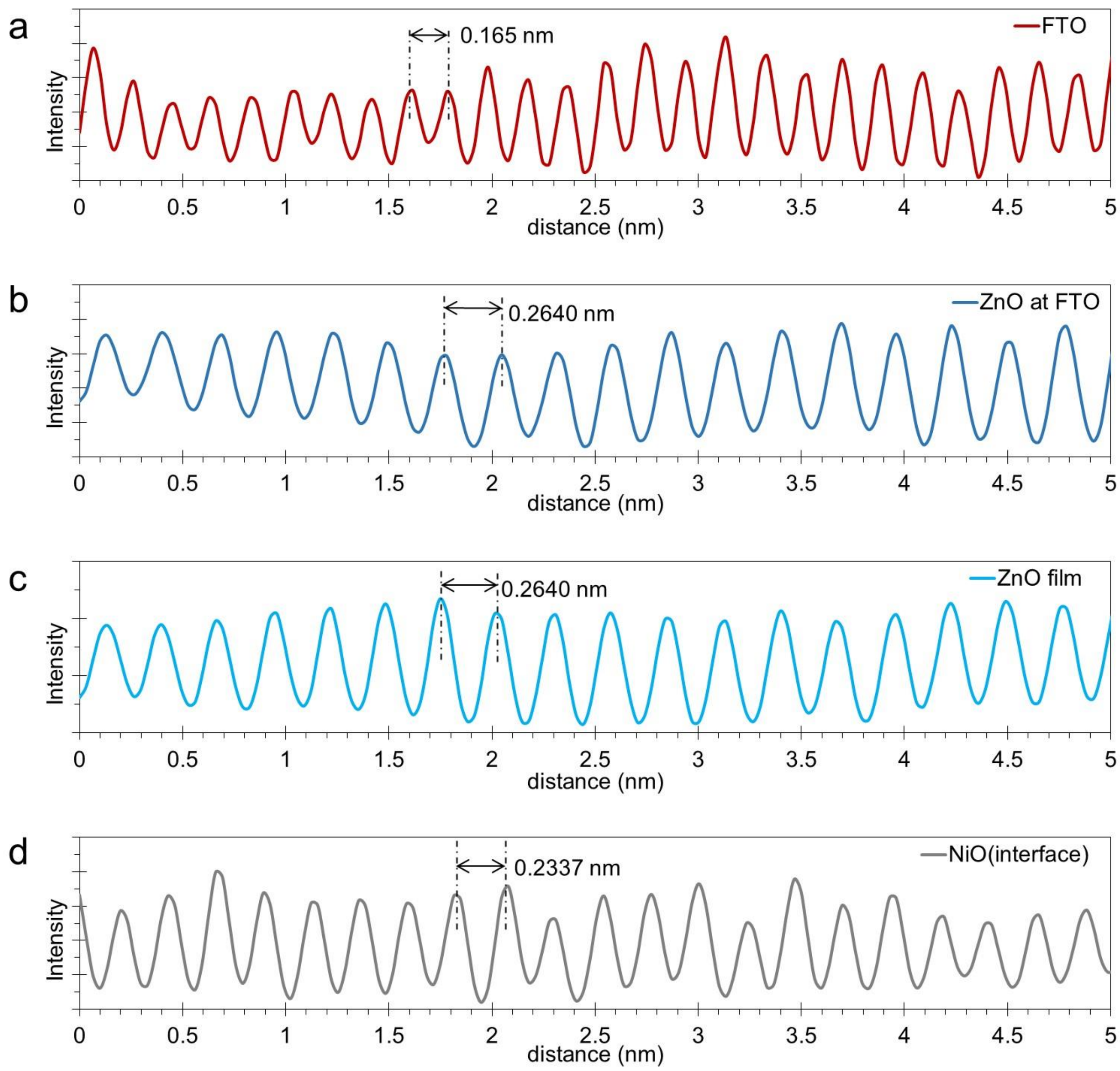

Supplementary Fig. 3 | (a) Intensity line profile of F:SnO_2 (FTO) for the region marked in Fig. 2 b. (b) Intensity line profile of ZnO at FTO interface for the region marked in Fig. 2b. (c) Intensity line profile of ZnO film for the region marked in Fig. 2c. (d) Intensity line profile of NiO at ZnO interface for the region marked in Fig. 2d.

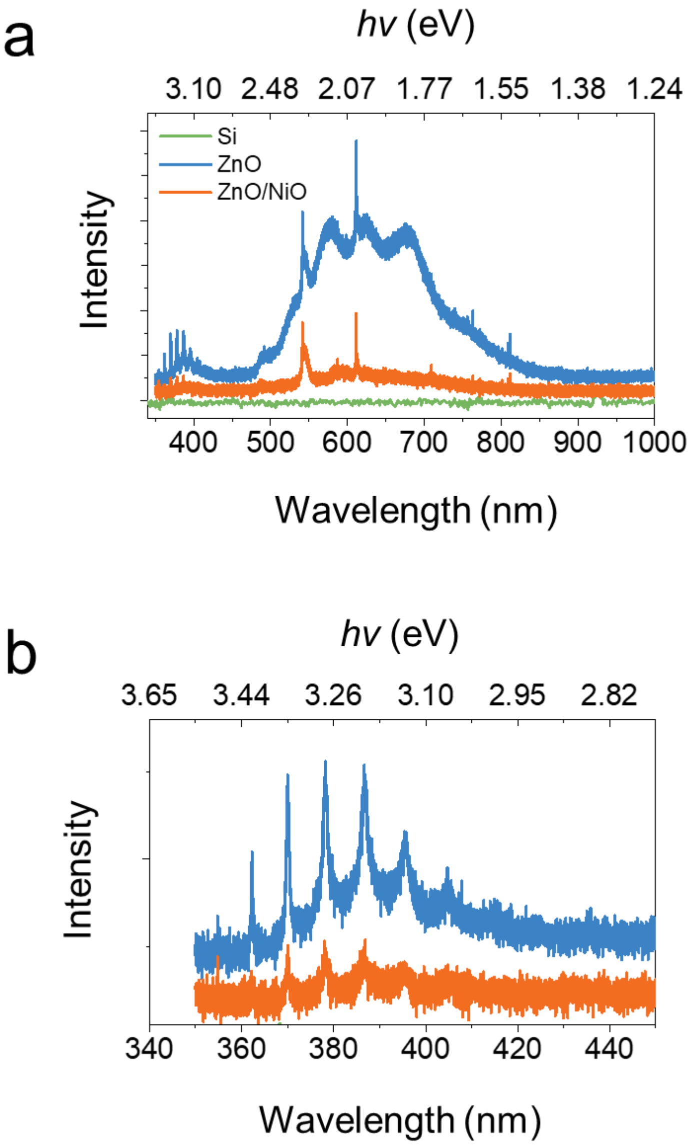

Supplementary Fig. 4 | (a) Photoluminescence spectra of Si, ZnO, and ZnO/NiO with an excitation wavelength of 355 nm. (b) Photoluminescence spectra of ZnO (blue) and ZnO/NiO (orange) correspond to high energy photon wavelength from 340 nm to 450 nm.

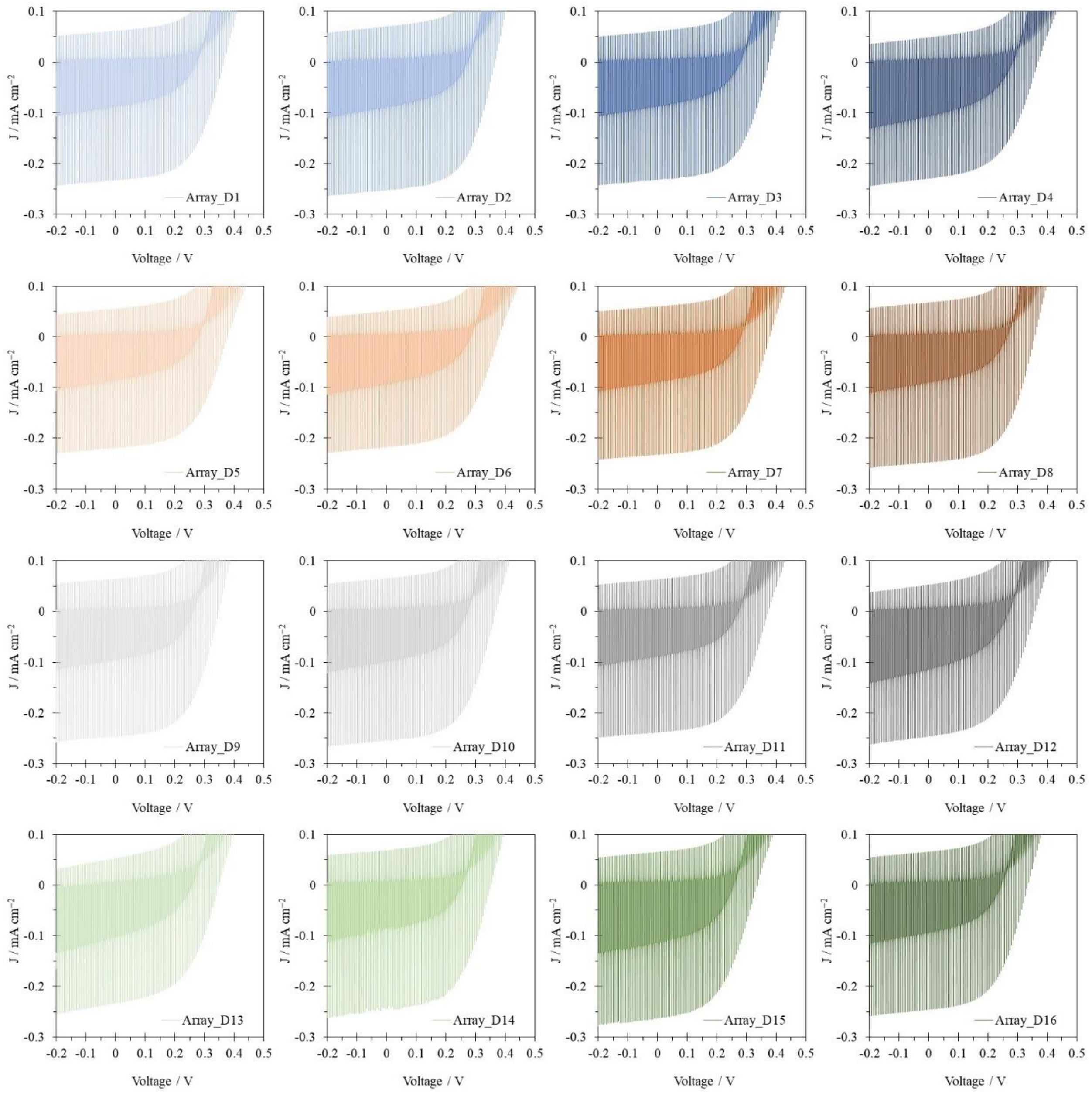

Supplementary Fig. 5 | Current-voltage characteristics of the TPV array devices. The array of 4 × 4 has 16 devices (D1-D16). A pulsed illumination wavelength of 365 nm and intensity of 0.5 mW cm^{-2} was used. The scan speed and sample interval were 0.35 V s^{-1} and 3 μV, respectively.

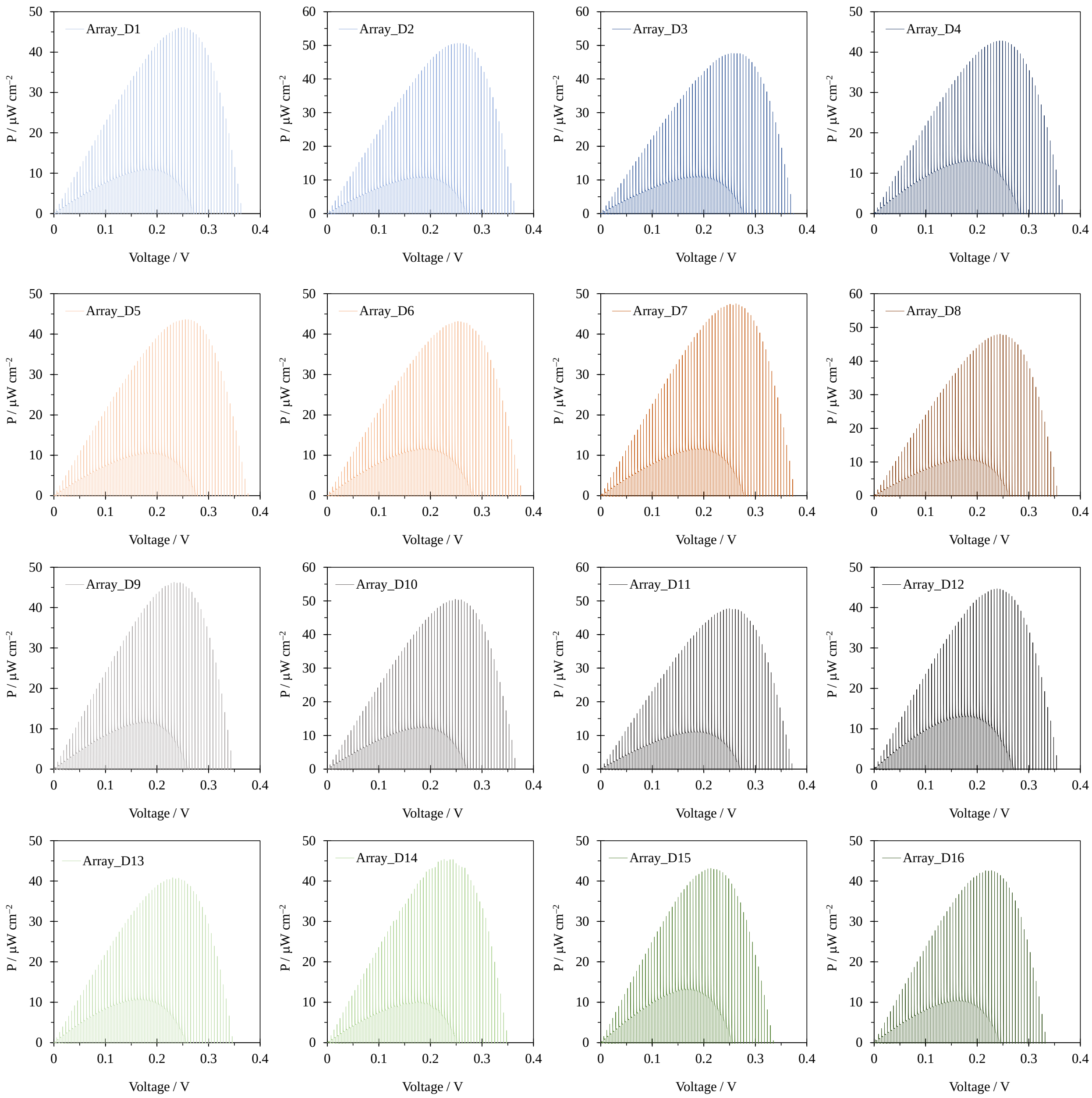

Supplementary Fig. 6 | Power-voltage characteristics of the TPV array devices obtained from the I-V characteristic shown in Supplementary Fig 5. These plots show output power for load voltage. The peak value of the power plot shows the maximum output power and, hence, maximum voltage and current.

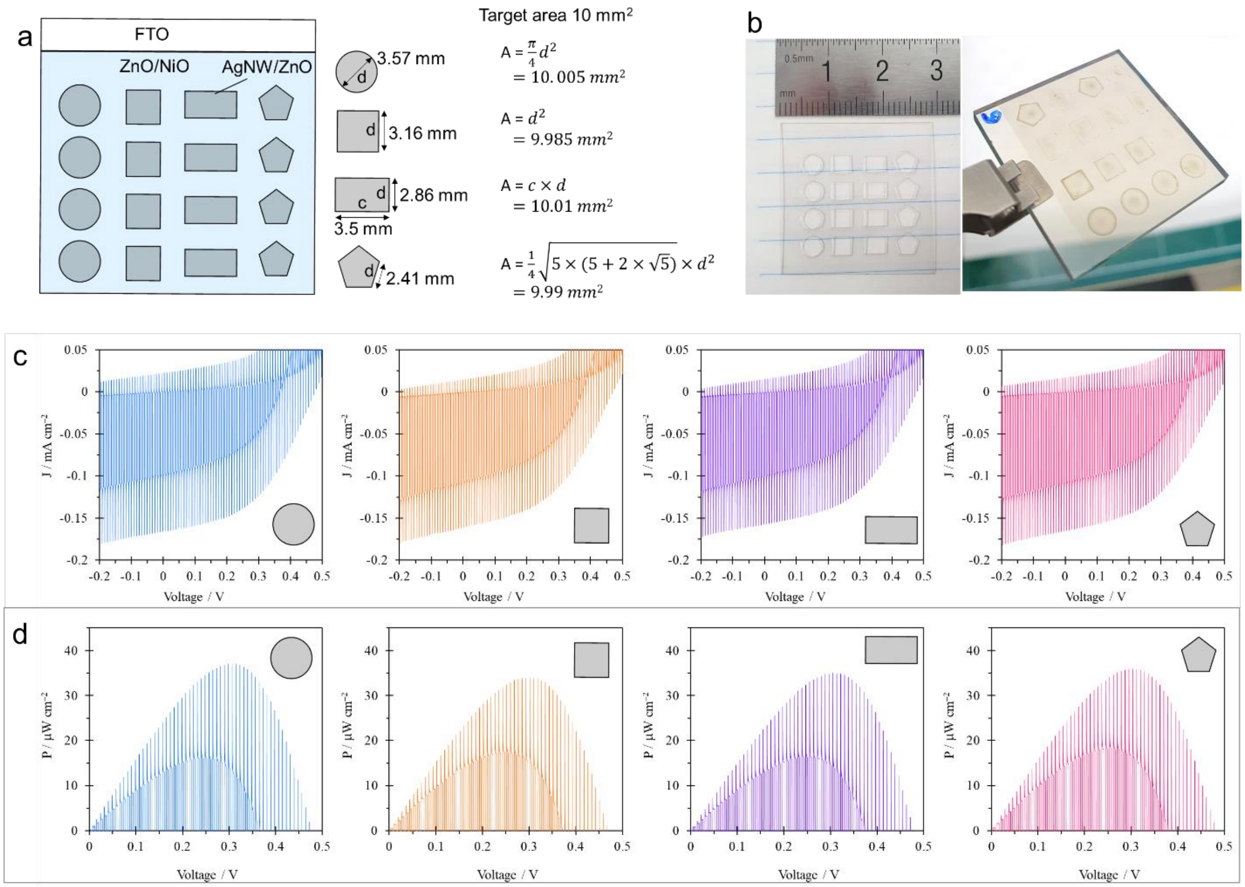

Supplementary Fig. 7 | (a) Schematic showing the mask design of the top electrode array of circular, square, rectangular, and pentagon shapes with a target area of 10 mm^2. (b) Original photo-image of the mask fabricated using the laser patterning and THPD array with various shapes of the top electrode. (c) Current density-voltage and (d) power density-voltage characteristic plot of the devices with various shapes of the top electrode. The pulsed frequency of 60 Hz, duty cycle of 50%, scan speed of 0.5 V s^{-1}, sample interval of 10 μV, illumination wavelength of 365 nm, and intensity of 400 μW cm^{-2} were set during the measurements.

Supplementary Table 2 | Summary of the performance parameters of the transparent pyroelectric heterojunction device with AgNW/ZnO top electrode of circular, square, rectangular, and pentagon shapes with identical areas.

Parameters	Top electrode shape of area 10 mm^2							
	Circular		Square		Rectangular		Pentagon	
	PV	PE-PV	PV	PE-PV	PV	PE-PV	PV	PE-PV
J_{SC} ($mA\,cm^{-2}$)	0.0977	0.1661	0.103	0.1614	0.0994	0.1576	0.1085	0.1656
V_{OC} (V)	0.366	0.481	0.372	0.468	0.374	0.4795	0.3799	0.486
P_{max} ($\mu W\,cm^{-2}$)	16.22	37.05	17.4	33.91	16.45	35.06	18.34	35.97
IPCE (%)	82.98	141.07	87.48	137.08	84.42	133.85	92.15	140.65
FF (%)	45.36	46.38	45.41	44.89	44.26	46.39	44.49	44.69
PCE (%)	4.06	9.26	4.35	8.48	4.11	8.77	4.58	8.99

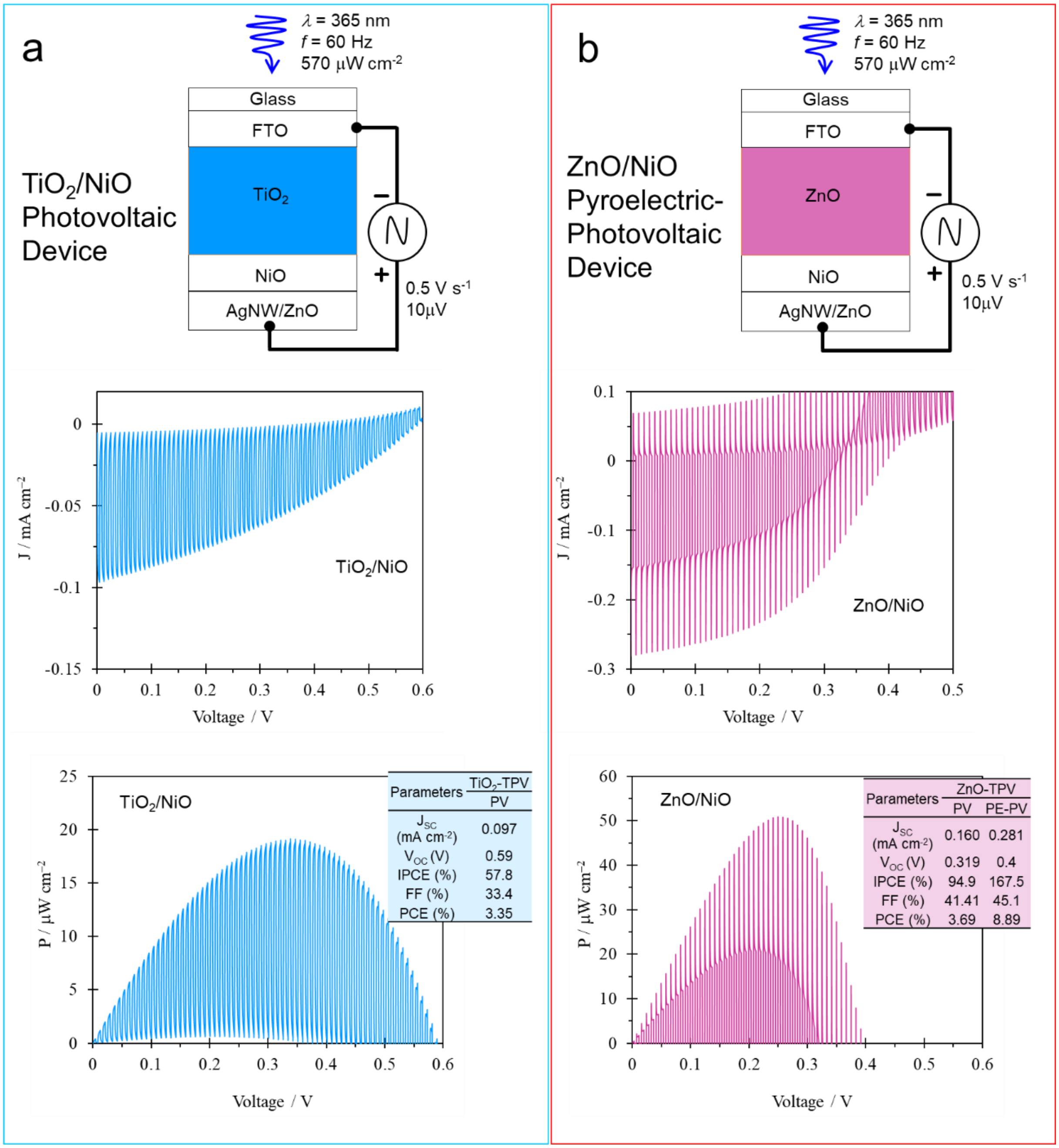

Supplementary Fig. 8 | Distinguish the current, voltage, and output power of PE and PV components using the centrosymmetric and non-centrosymmetric absorbers. Device schematic, J-V and P-V characteristic plots of the heterojunction device of (a) TiO_2-anatase and (b) ZnO absorbers. Pulsed illumination has a duty cycle of 50, frequency of 60 Hz, and intensity of 570 μW cm^{-2}. The performance parameters of both devices are summarized in the inset in the P-V characteristic plot.

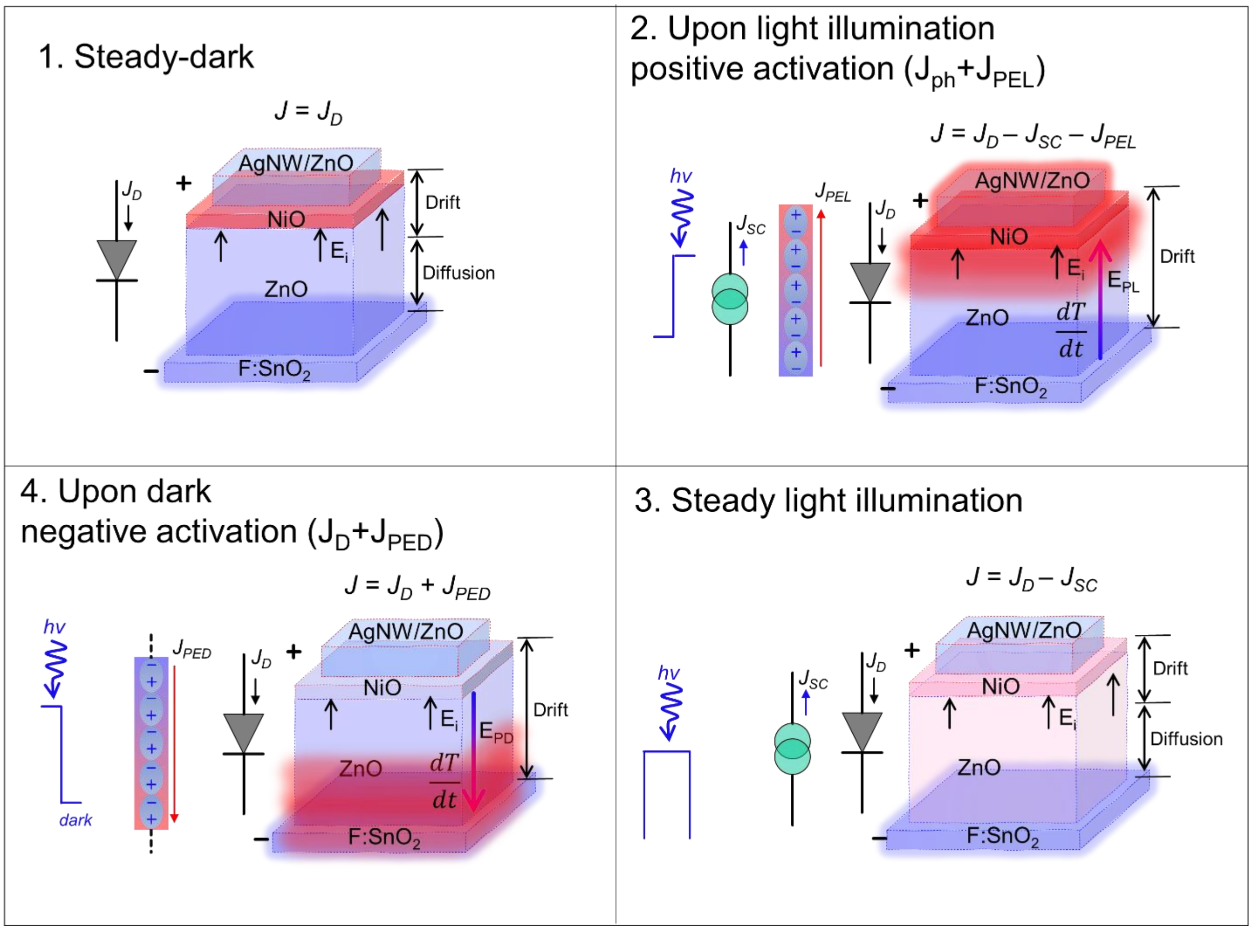

Supplementary Fig. 9 | A schematic of the device and its built-in electric field (E_i) and the pyroelectric field (E_{PL} in the light-on state and E_{PD} in the dark state). The schematics demonstrate how drift and diffusion transport are modulated under pulsed light illuminations. The reference panels illustrate electric field directions and current flow directions for 1) steady-dark, 2) upon light illumination, 3) steady-light illumination, and 4) upon dark states.

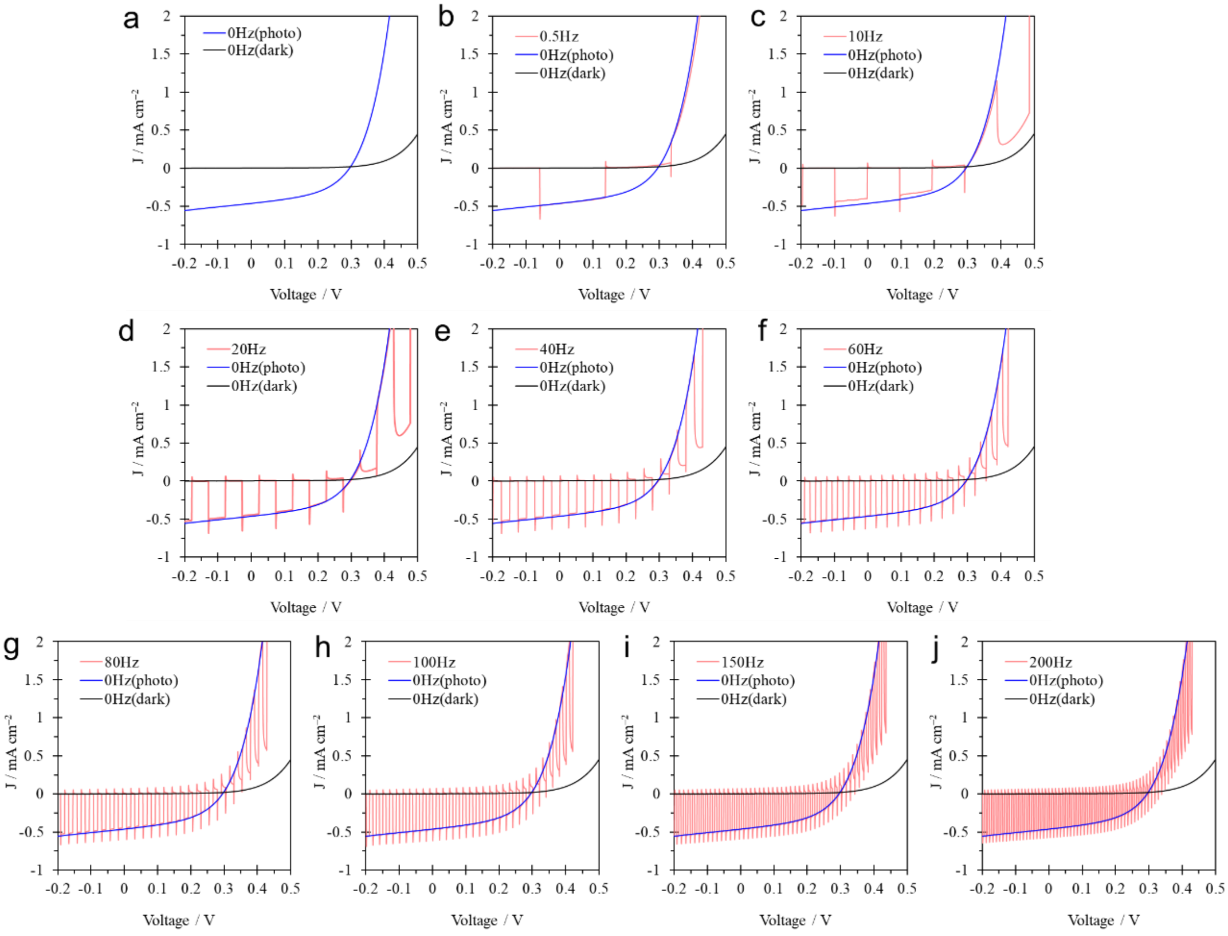

Supplementary Fig. 10 | Current density-voltage characteristics of the TPHD PV device for various pulsed frequencies of light illumination with wavelengths of 365 nm and intensity of 2 mW cm^{-2}. (a) 0 Hz, (b) 0.5 Hz, (c) 10 Hz, (d) 20 Hz, (e) 40 Hz, (f) 60 Hz, (g) 80 Hz, (h) 100 Hz, (i) 150 Hz, (j) 200 Hz. Throughout the measurements, scan speed and sample interval were 2 V s^{-1} and 100 μV, respectively.

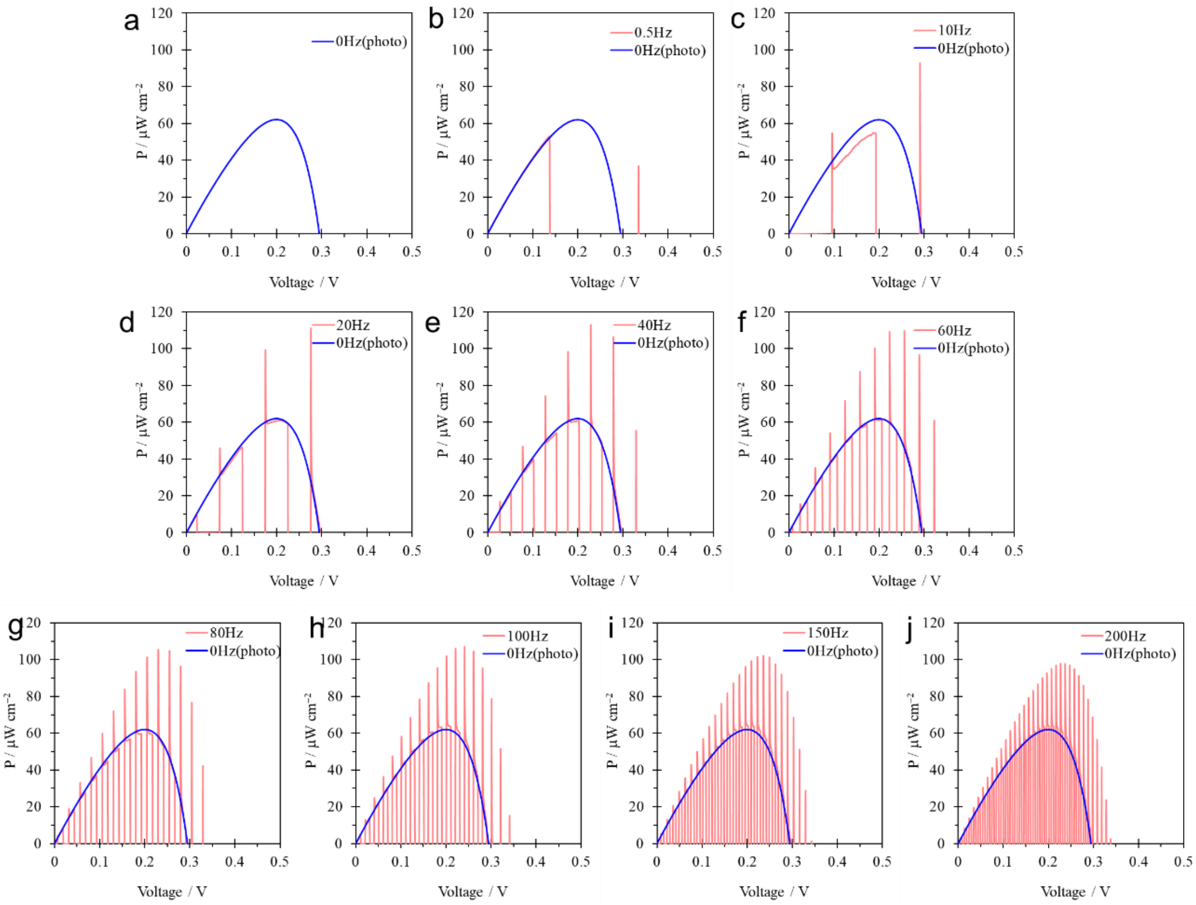

Supplementary Fig. 11 | Power density-voltage characteristics of the TPHD device for various pulsed frequencies of light illumination with wavelengths of 365 nm and intensity of 2 mW cm^{-2}. (a) 0 Hz, (b) 0.5 Hz, (c) 10 Hz, (d) 20 Hz, (e) 40 Hz, (f) 60 Hz, (g) 80 Hz, (h) 100 Hz, (i) 150 Hz, (j) 200 Hz. Throughout the measurements, scan speed and sample interval were 2 V s^{-1} and 100 μV, respectively. These plots show output power for load voltage. The peak value of the power plot shows the maximum output power and, hence, maximum voltage and current density.

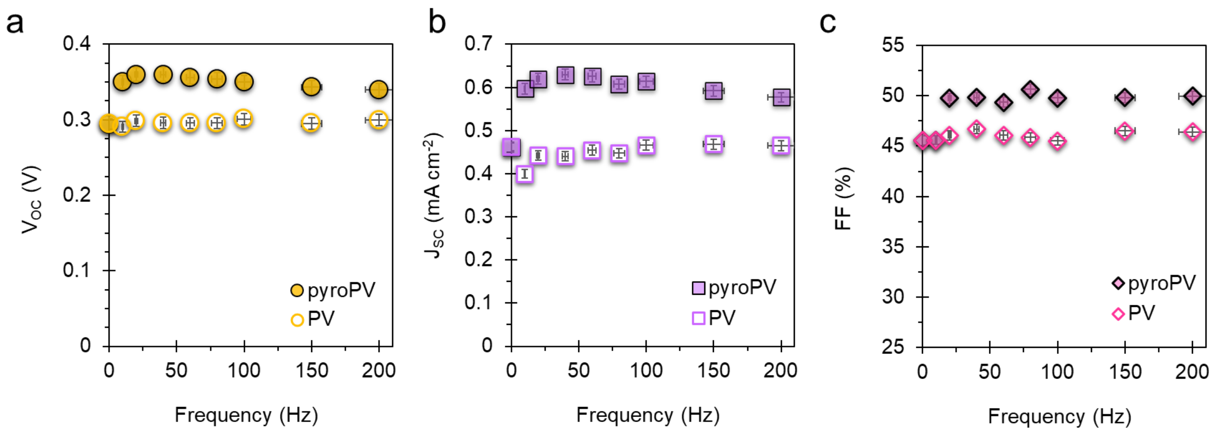

Supplementary Fig. 12 | Summary of the performance parameters of the TPHD device as a function of pulse illumination frequency (*f*). (a) V_{OC} vs. *f*. (b) J_{SC} vs. *f*, (c) FF vs. *f*. (error bar is 2.5%) These parameters were obtained from the J-V and P-V characteristic plots in Supplementary Fig. 10-11.

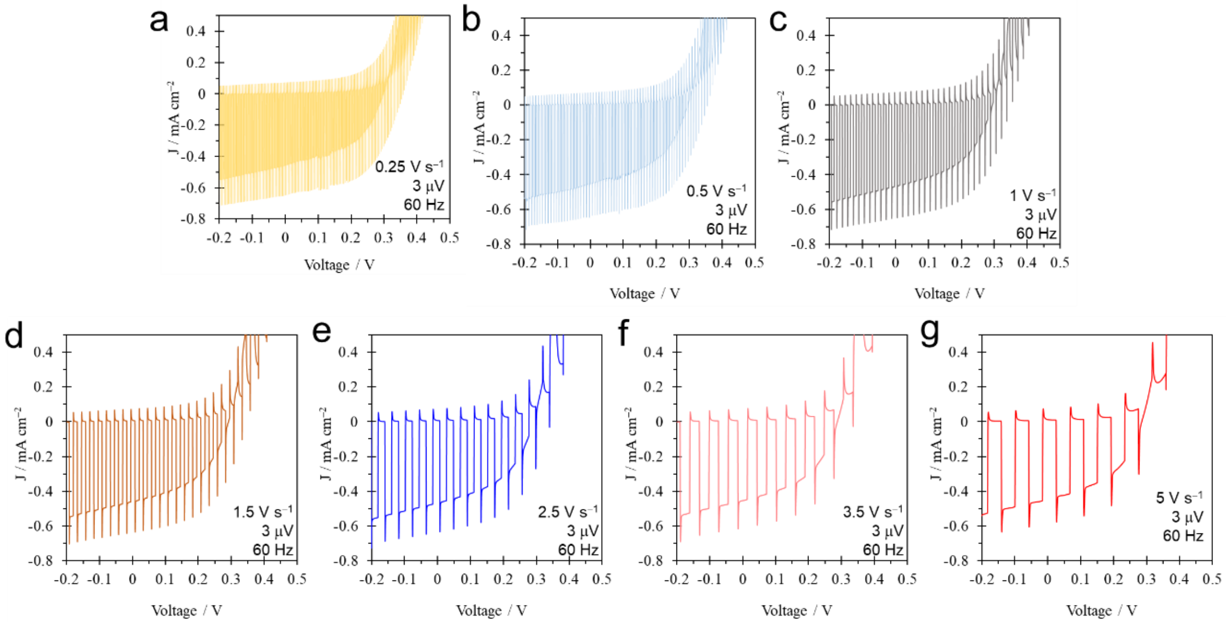

Supplementary Fig. 13 | Current density-voltage characteristics of the TPHD device for various scan rates. (a) 0.25 V s^{-1}, (b) 0.5 V s^{-1}, (c) 1 V s^{-1}, (d) 1.5 V s^{-1}, (e) 2.5 V s^{-1}, (f) 3.5 V s^{-1}, and (g) 5 V s^{-1}. The sample interval was 3 μV. The light illumination of the wavelength, intensity, and pulsed frequency was 365 nm, 2 mW cm^{-2}, and 60 Hz, respectively.

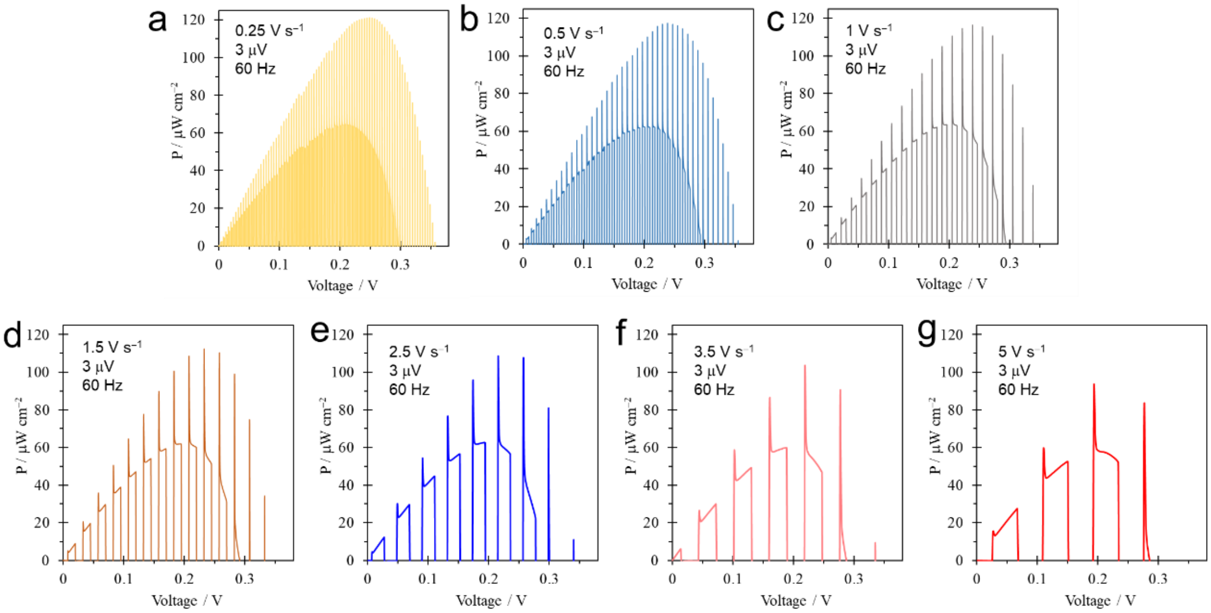

Supplementary Fig. 14 | Power density-voltage characteristics of the TPHD device for various scan rates. (a) 0.25 V s^{-1}, (b) 0.5 V s^{-1}, (c) 1 V s^{-1}, (d) 1.5 V s^{-1}, (e) 2.5 V s^{-1}, (f) 3.5 V s^{-1}, and (g) 5 V s^{-1}. The sample interval was 3 μV. The light illumination of the wavelength, intensity, and pulsed frequency was 365 nm, 2 mW cm^{-2}, and 60 Hz, respectively. These plots show output power for load voltage. The peak value of the power plot shows the maximum output power and, hence, maximum voltage and current density.

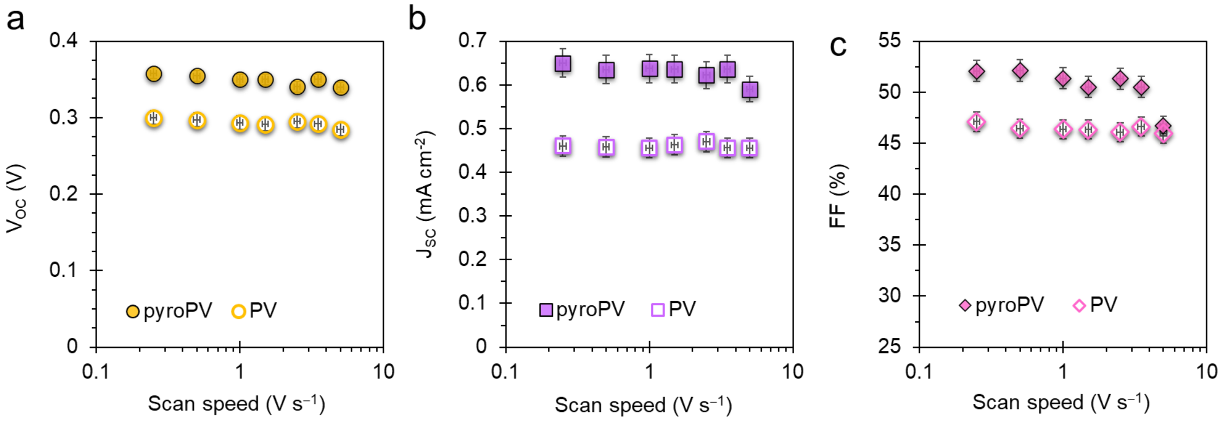

Supplementary Fig. 15 | Summary of the performance parameters of the TPHD device as a function of the scan speed of current-voltage characteristics. (a) V_{OC}, (b) J_{SC}, and (c) FF versus scan speed in V s^{-1} (error bar is 5%). These parameters were obtained from the J-V and P-V characteristic plots in Supplementary Fig. 13-14.

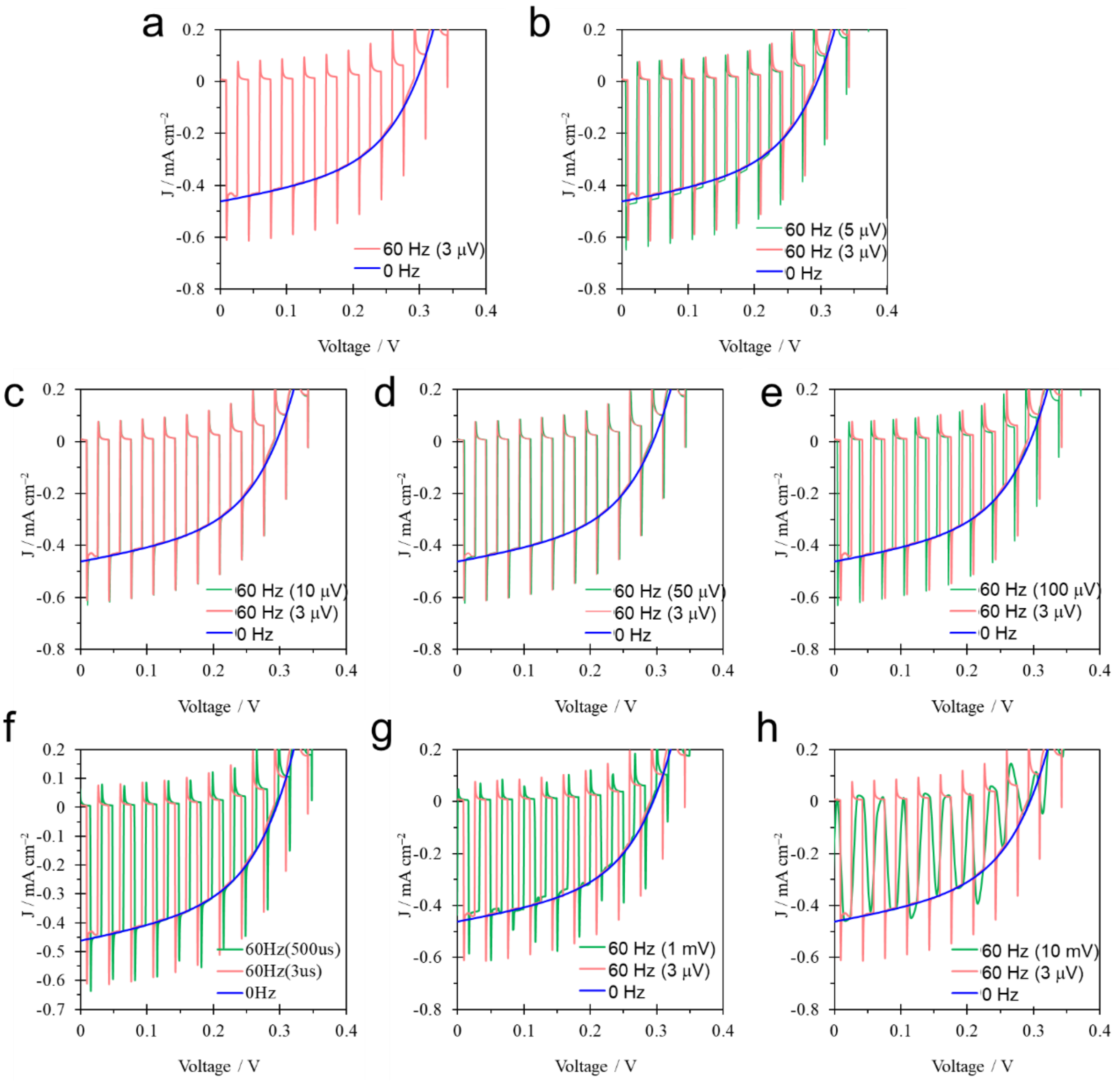

Supplementary Fig. 16 | Current density-voltage characteristics of the TPHD device for various sample intervals. (a) 3 μV, (b) 5 μV, (c) 10 μV, (d) 50 μV, (e) 100 μV, (f) 500 μV, (g) 1 mV, and (h) 10 mV. Throughout these measurements, scan speed was 2 V s^{-1}. The light illumination of the wavelength, intensity, and pulsed frequency was 365 nm, 2 mW cm^{-2}, and 60 Hz, respectively.

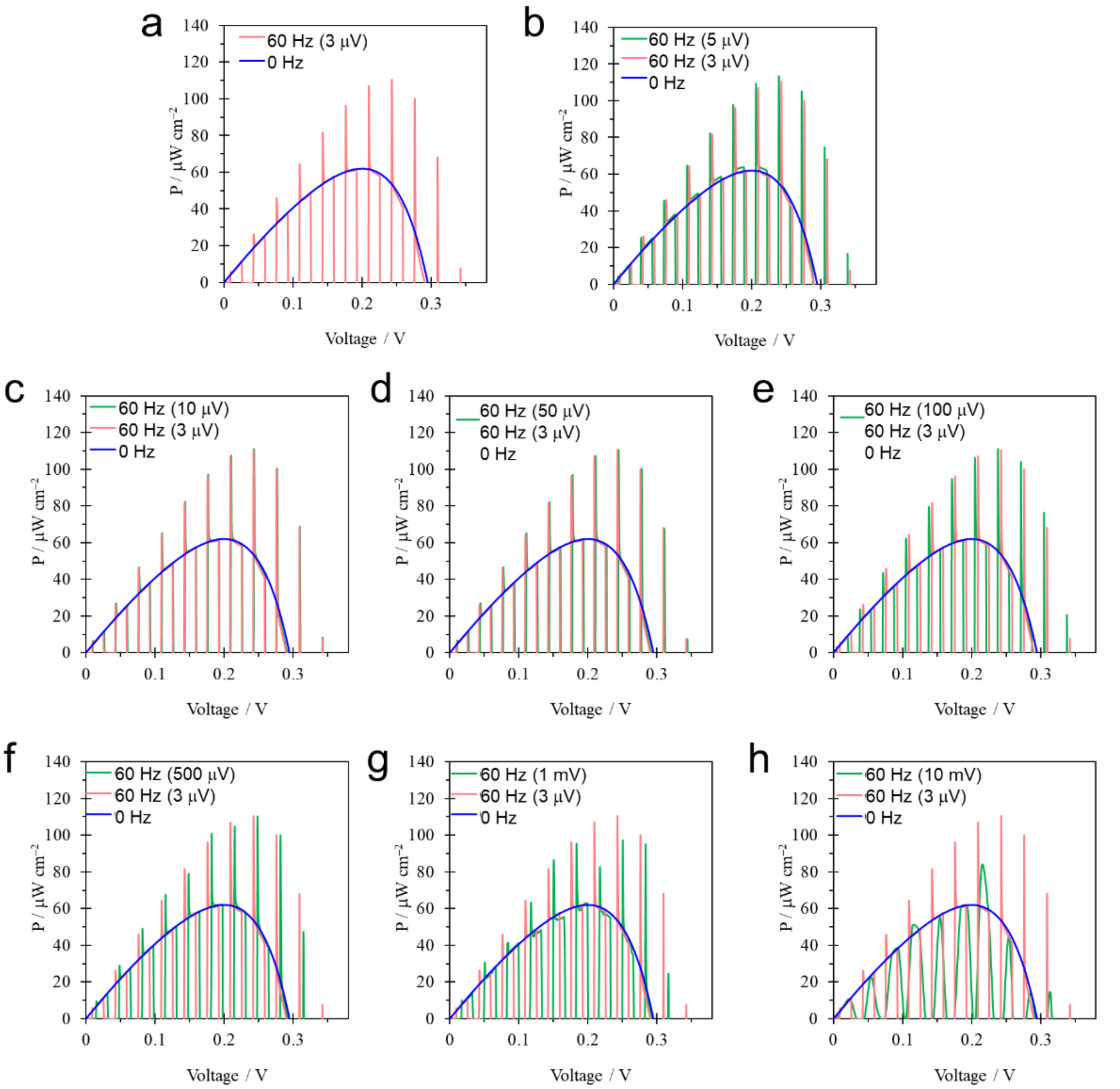

Supplementary Fig. 17 | Power-voltage characteristics of the TPHD device for various sample intervals. (a) 3 μV, (b) 5 μV, (c) 10 μV, (d) 50 μV, (e) 100 μV, (f) 500 μV, (g) 1 mV, and (h) 10 mV. Throughout these measurements, scan speed was 2 V s^{-1}. The light illumination of the wavelength, intensity, and pulsed frequency was 365 nm, 2 mW cm^{-2}, and 60 Hz, respectively. These plots show output power for load voltage. The peak value of the power plot shows the maximum output power and, hence, maximum voltage and current density.

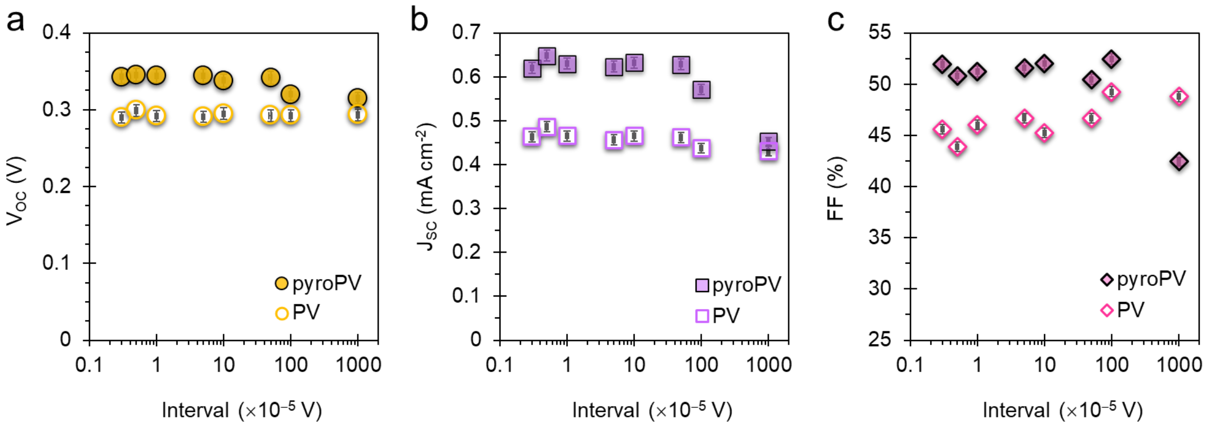

Supplementary Fig. 18 | Summary of the performance parameters of the TPHD device as a function of the sample interval of current density-voltage characteristics. (a) V_{OC}, (b) J_{SC}, and (c) FF versus sample interval in $\times 10^{-5}$ V (error bar is 5%). These parameters were obtained from the J-V and P-V characteristic plots shown in Supplementary Fig. 16-17.

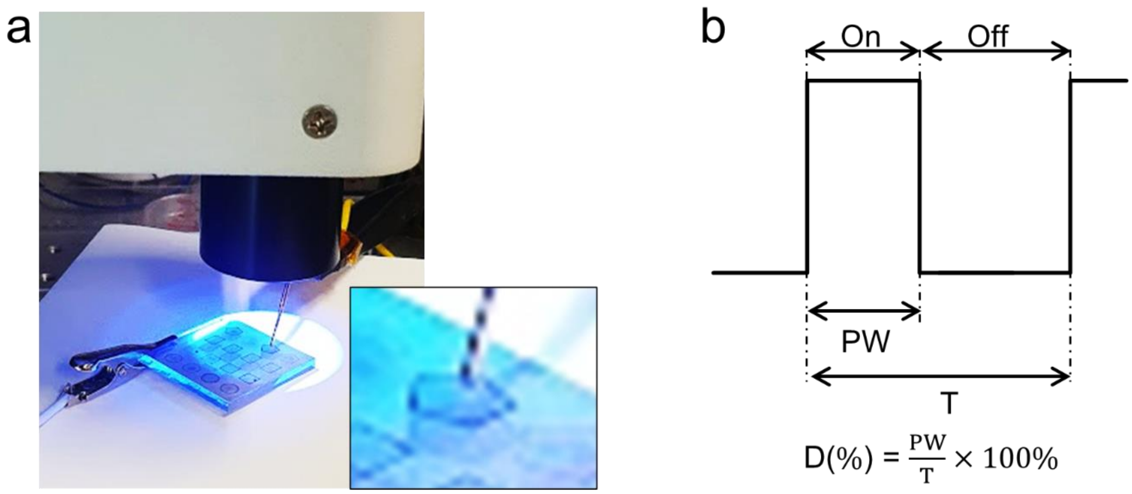

c

Duty cycle D (%)	Pulse width PW (ms)	Off time (ms)	Total signal period T (ms)	Photon density (cm^{-2} s^{-1})	Photon dose per cycle (cm^{-2})	Expected photocurrent density ($\mu A/cm^2$)
2	0.4	19.6	20	3.48831×10^{15}	1.395×10^{12}	11.17
10	2	18			6.977×10^{12}	55.88
20	4	16			1.395×10^{13}	111.77
30	6	14			2.093×10^{13}	167.66
40	8	12			2.790×10^{13}	223.55
50	10	10			3.488×10^{13}	279.44
60	12	8			4.186×10^{13}	335.33
70	14	6			4.884×10^{13}	391.22
80	16	4			5.581×10^{13}	447.11
90	18	2			6.279×10^{13}	503.00
98	19.6	0.4			6.837×10^{13}	547.71
100	20	0			6.977×10^{13}	558.89

Supplementary Fig. 19 | Experimental design on clarification on the pyroelectric power evaluation indicators. (a) The device under test incorporates the pulse on/off ratio employing a duty cycle. (b) Schematic showing the duty cycle calculation. (c) Summary of duty cycle, pulse width, photon density, and dose during complete pulse interval.

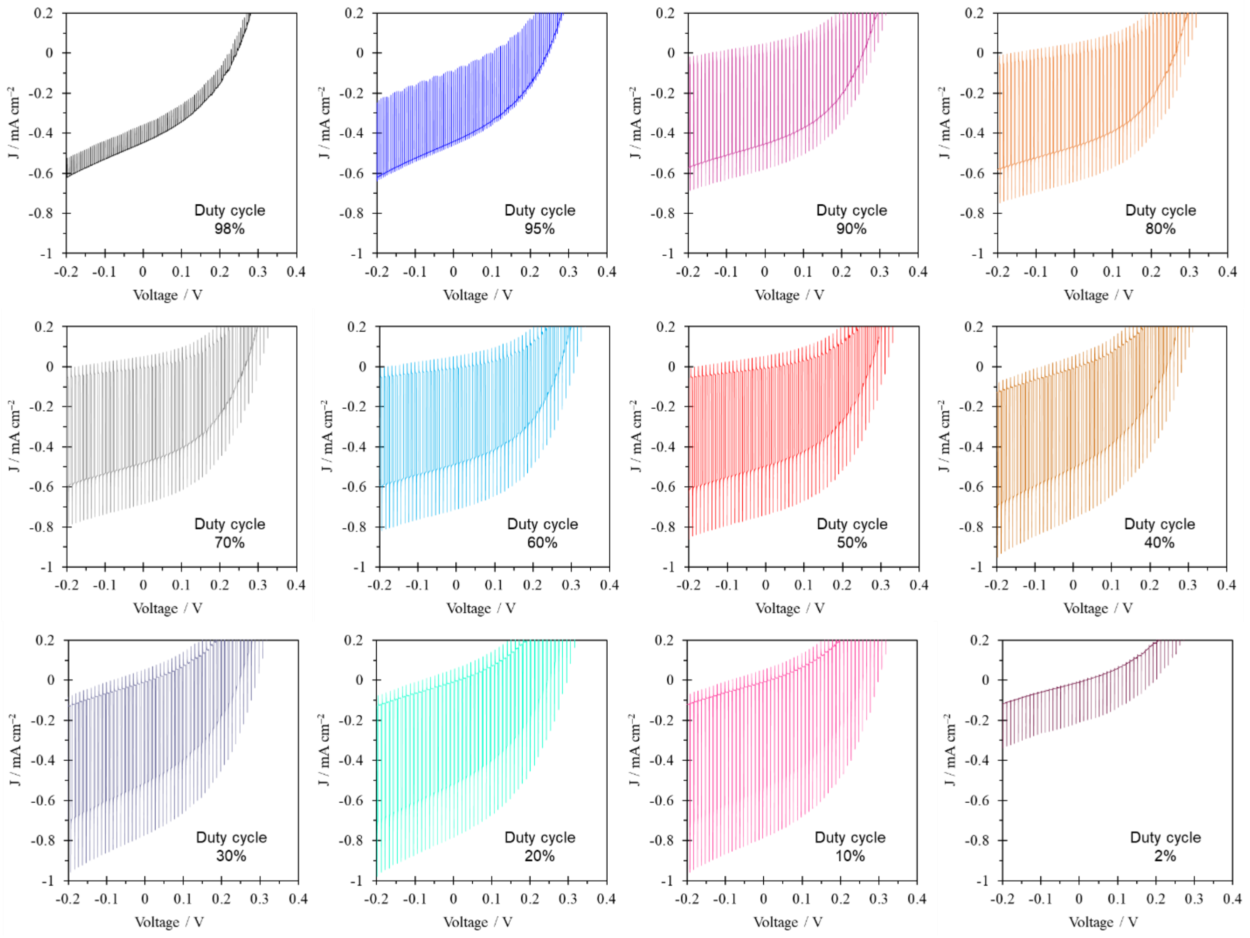

Supplementary Fig. 20 | Current density-voltage (J-V) characteristics of the device under the pulsed light illumination of various duty cycles from 2% to 98%. Throughout these measurements, scan speed was 0.5 V s^{-1}. The light illumination of the wavelength, intensity, and pulsed frequency was 365 nm, 1.9 mW cm^{-2}, and 50 Hz, respectively.

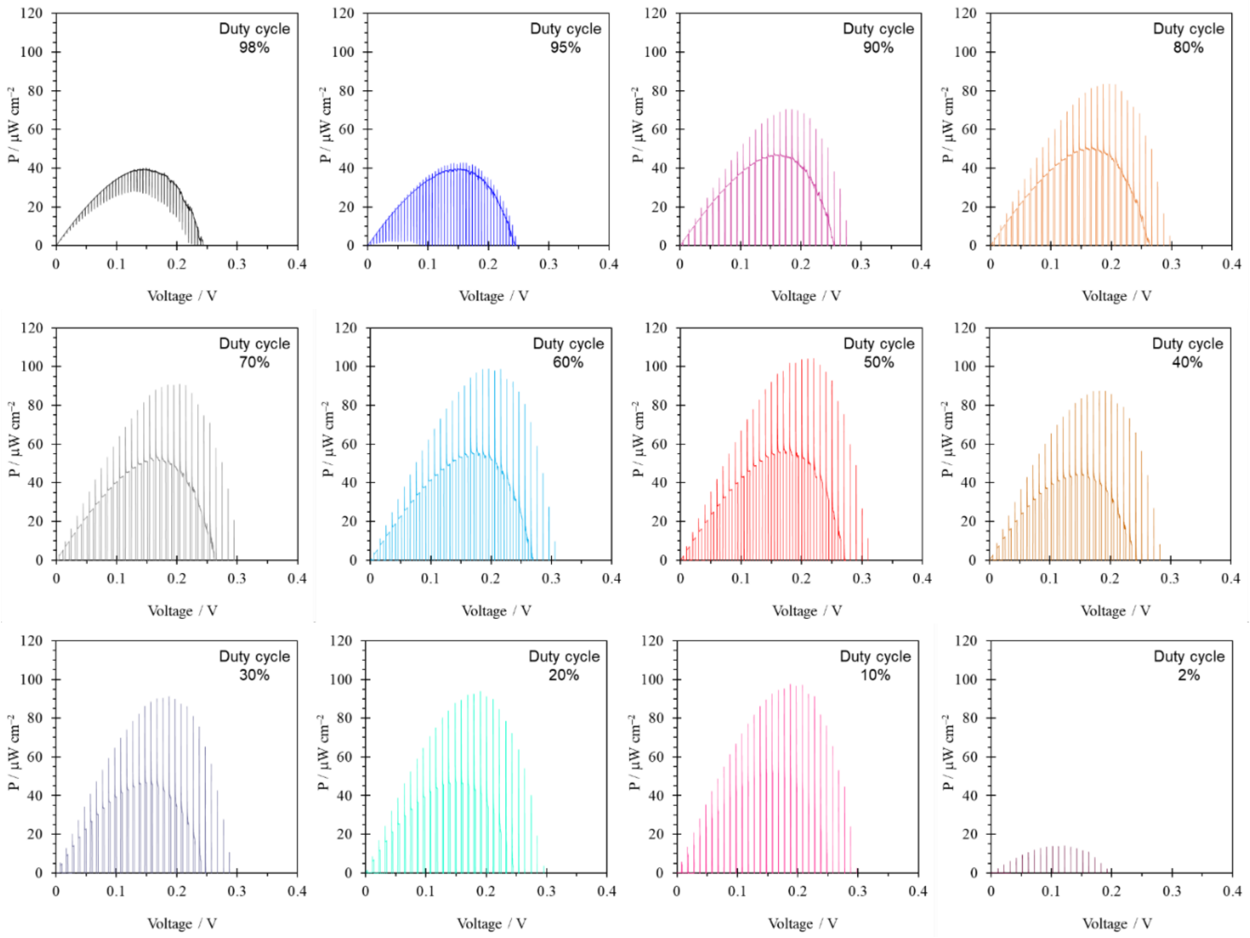

Supplementary Fig. 21 | Power density-voltage (P-V) characteristics of the device under the pulsed light illumination of various duty cycles from 2% to 98%. Throughout these measurements, the scan speed was 0.5 V s^{-1}. The light illumination of the wavelength, intensity, and pulsed frequency was 365 nm, 1.9 mW cm^{-2}, and 50 Hz, respectively. These plots show output power for load voltage. The peak value of the power plot shows the maximum output power and, hence, maximum voltage and current density.

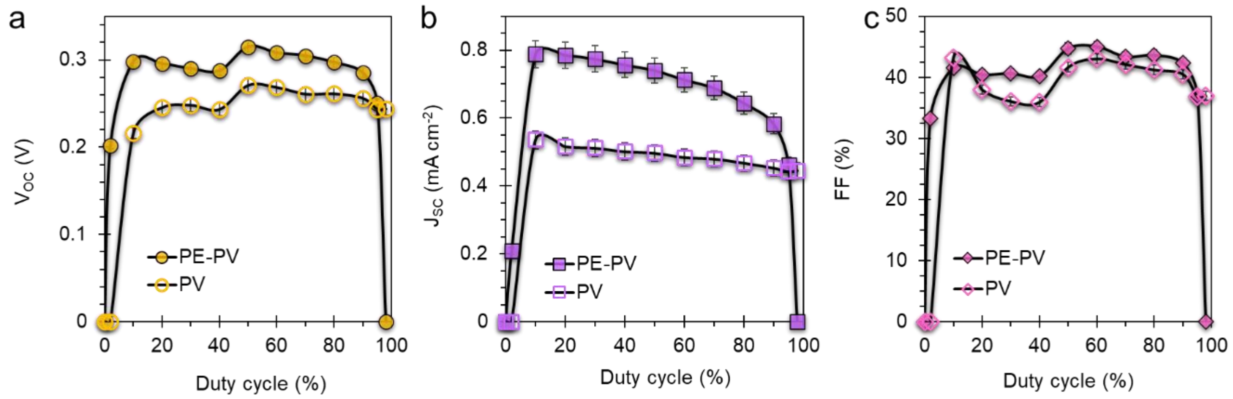

Supplementary Fig. 22 | Summary of the performance parameters of the TPHD device as a function duty cycle of the pulse illumination. (a) V_{OC}, (b) J_{SC}, and (c) FF versus duty cycle in % (error bar is 2.5%). These parameters were obtained from the J-V and P-V characteristic plots shown in Supplementary Fig. 20-21.

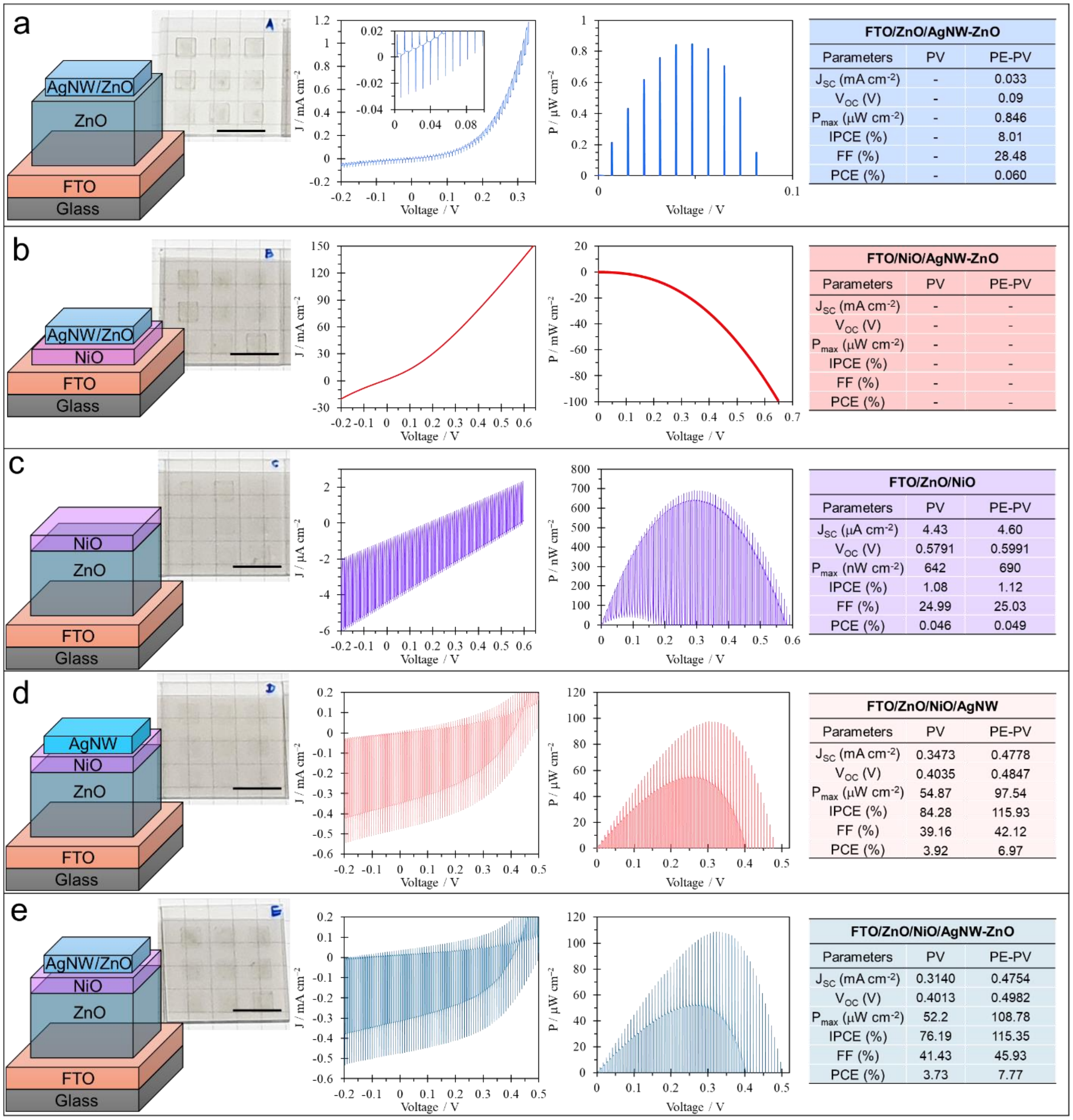

Supplementary Fig. 23 | Assessment of the device structure to enable pyroelectric-photovoltaic phenomena. (a) Glass/FTO/ZnO/AgNW/ZnO. (b) Glass/FTO/NiO/AgNW/ZnO. (c) Glass/FTO/ZnO/NiO. (d) Glass/FTO/ZnO/NiO/AgNW. (e) Glass/FTO/ZnO/NiO/AgNW/ZnO. Each panel summarizes the device schematic, photo-image of the device (scale bar, 1cm), current density-voltage (J-V) and power density-voltage (P-V) characteristics, and performance parameters, including J_{SC}, V_{OC}, P_{max}, IPCE, FF, and PCE for PV and PE-PV. The wavelength, intensity, pulsed frequency, and duty cycle of light illumination were 365 nm, 1.4 mW cm^{-2}, 60 Hz, and 50%, respectively. Throughout these measurements, scan speed, and sample interval were 0.5 V s^{-1}, and 50 μV, respectively.

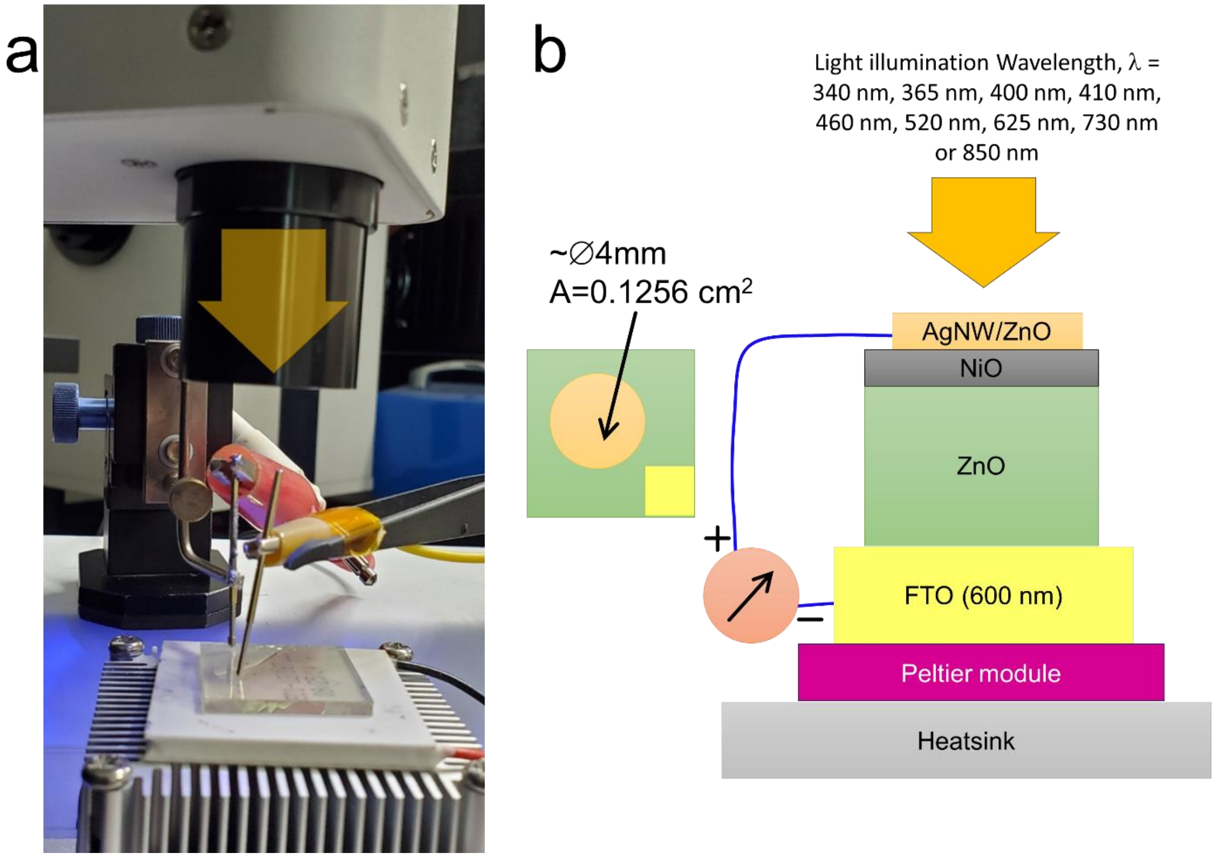

Supplementary Fig. 24 | (a) Measurement setup showing the pyro-photovoltaic device under test for spectral and thermal performances. The device was mounted on the Peltier module for thermal control under monochromatic pulse illumination. (b) The panel illustrates the connection of the device, the active area, light illumination wavelength, and thermal controller.

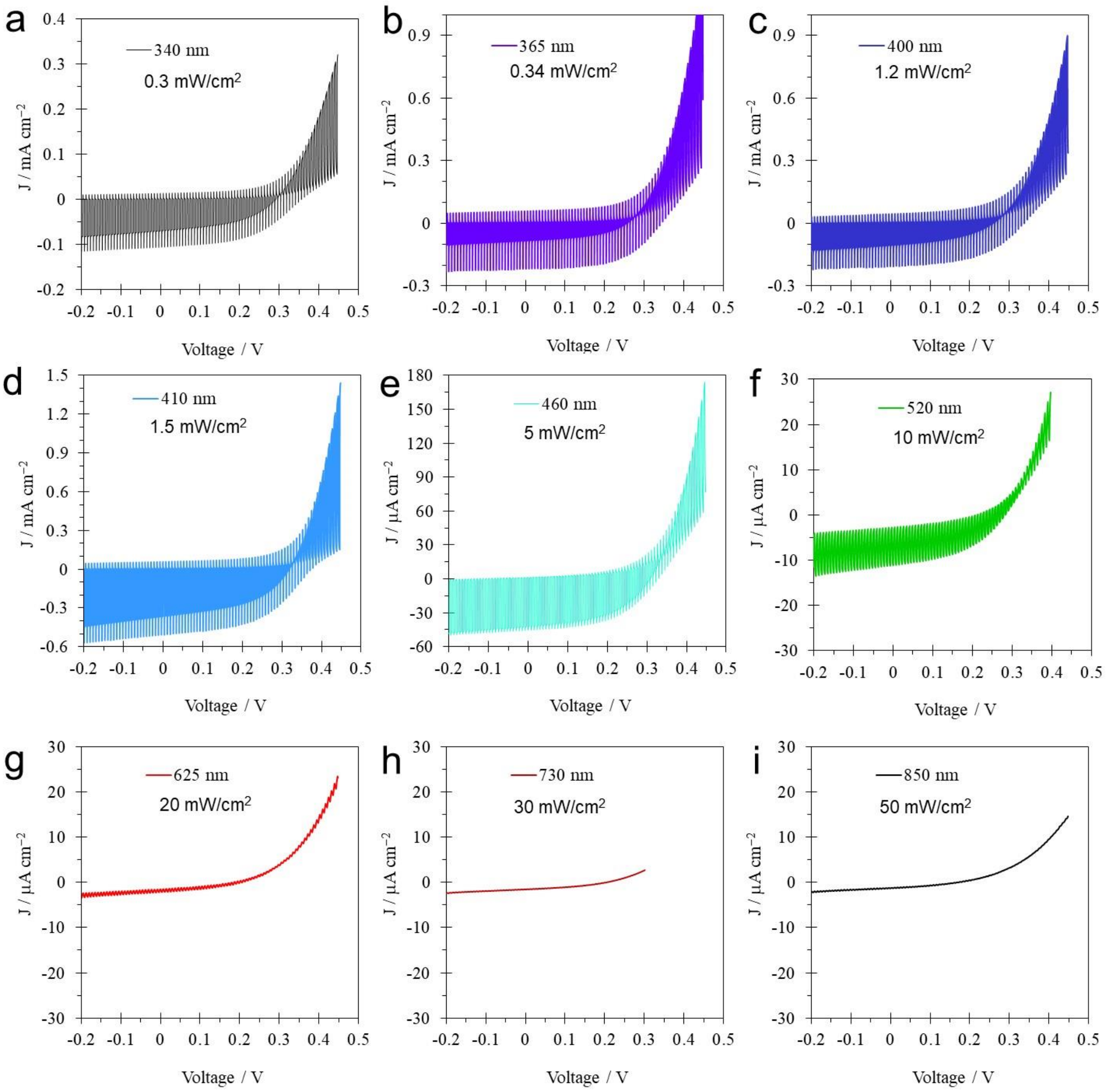

Supplementary Fig. 25 | Current-voltage characteristics of the TPV device under various wavelengths (λ) of light illumination. (a) λ of 340 nm and intensity of 0.3 mW cm^{-2}. (b) λ of 365 nm and intensity of 0.34 mW cm^{-2}. (c) λ of 400 nm and intensity of 0.1.2 mW cm^{-2}. (d) λ of 410 nm and intensity of 1.5 mW cm^{-2}. (e) λ of 460 nm and intensity of 5 mW cm^{-2}. (f) λ of 520 nm and intensity of 10 mW cm^{-2}. (g) λ of 625 nm and intensity of 20 mW cm^{-2}. (h) λ of 730 nm and intensity of 30 mW cm^{-2}. (i) λ of 850 nm and intensity of 50 mW cm^{-2}. Throughout these measurements, scan speed, sample interval, and illumination pulsed frequency were 0.5 V s^{-1}, 3 μV, and 60 Hz, respectively.

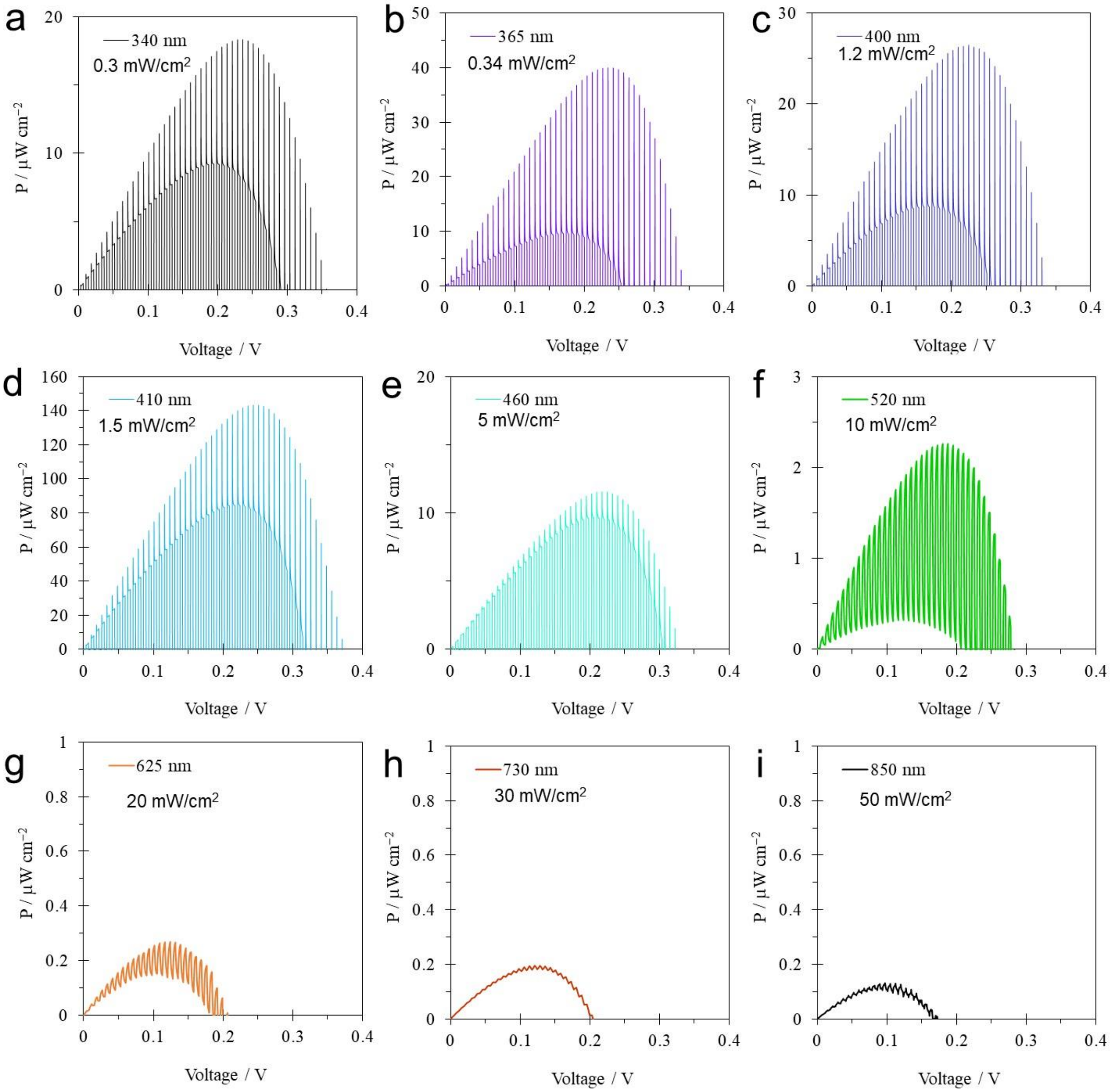

Supplementary Fig. 26 | Power-voltage characteristics of the TPV device under various wavelengths (λ) of light illumination. (a) λ of 340 nm and intensity of 0.3 mW cm^{-2}. (b) λ of 365 nm and intensity of 0.34 mW cm^{-2}. (c) λ of 400 nm and intensity of 0.1.2 mW cm^{-2}. (d) λ of 410 nm and intensity of 1.5 mW cm^{-2}. (e) λ of 460 nm and intensity of 5 mW cm^{-2}. (f) λ of 520 nm and intensity of 10 mW cm^{-2}. (g) λ of 625 nm and intensity of 20 mW cm^{-2}. (h) λ of 730 nm and intensity of 30 mW cm^{-2}. (i) λ of 850 nm and intensity of 50 mW cm^{-2}. Throughout these measurements, scan speed, sample interval, and illumination pulsed frequency were 0.5 V s^{-1}, 3 μV, and 60 Hz, respectively.

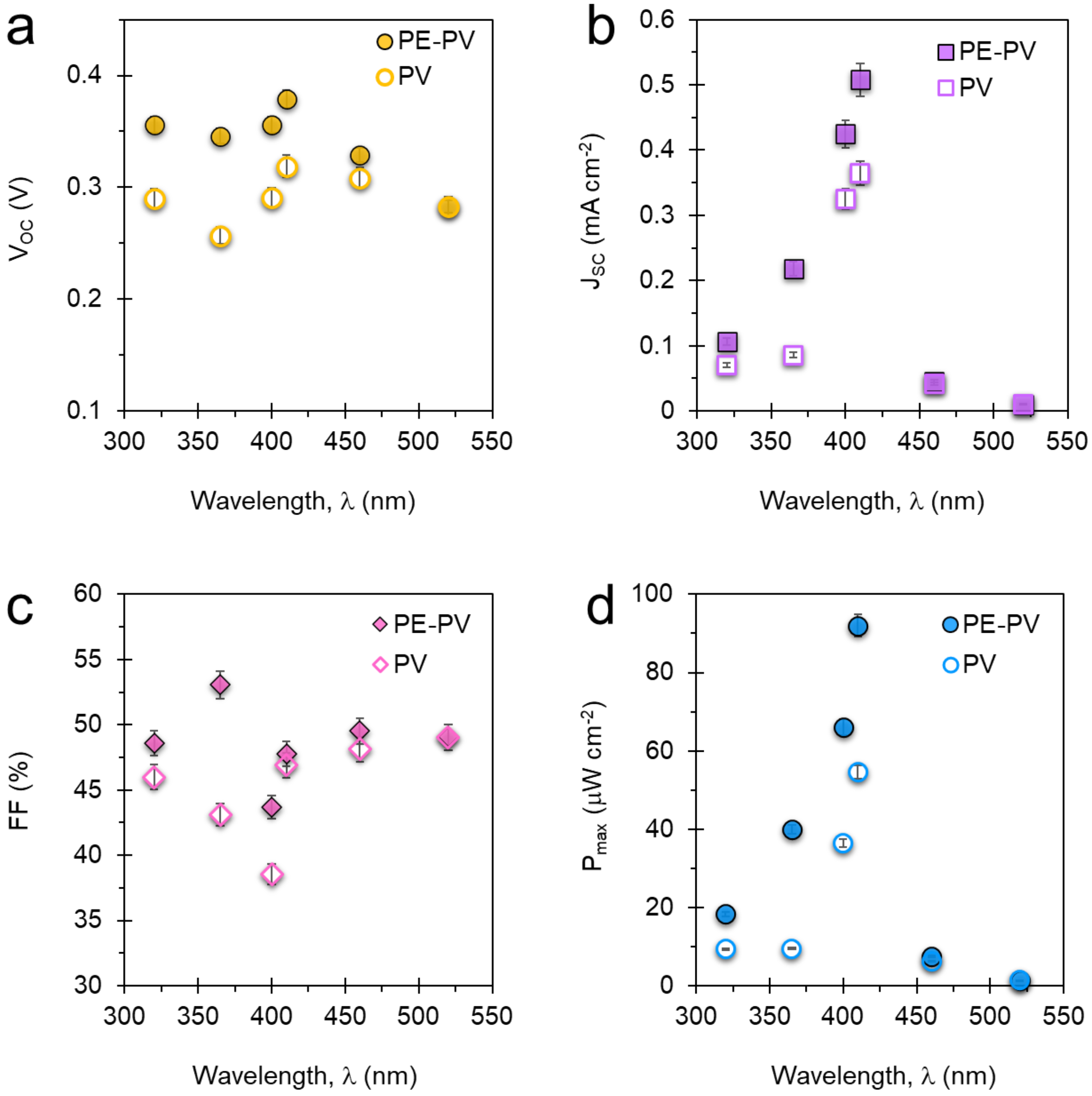

Supplementary Fig. 27 | Summary of the performance parameters of the pyro-TPV devices as a function of illumination wavelength. (a) V_{OC}, (b) J_{SC}, (c) FF, and (d) P_{max} as a function of wavelength (error bar is 5%). These parameters were obtained from the J-V characteristic and P-V plots shown in Supplementary Figs. 25 and 26.

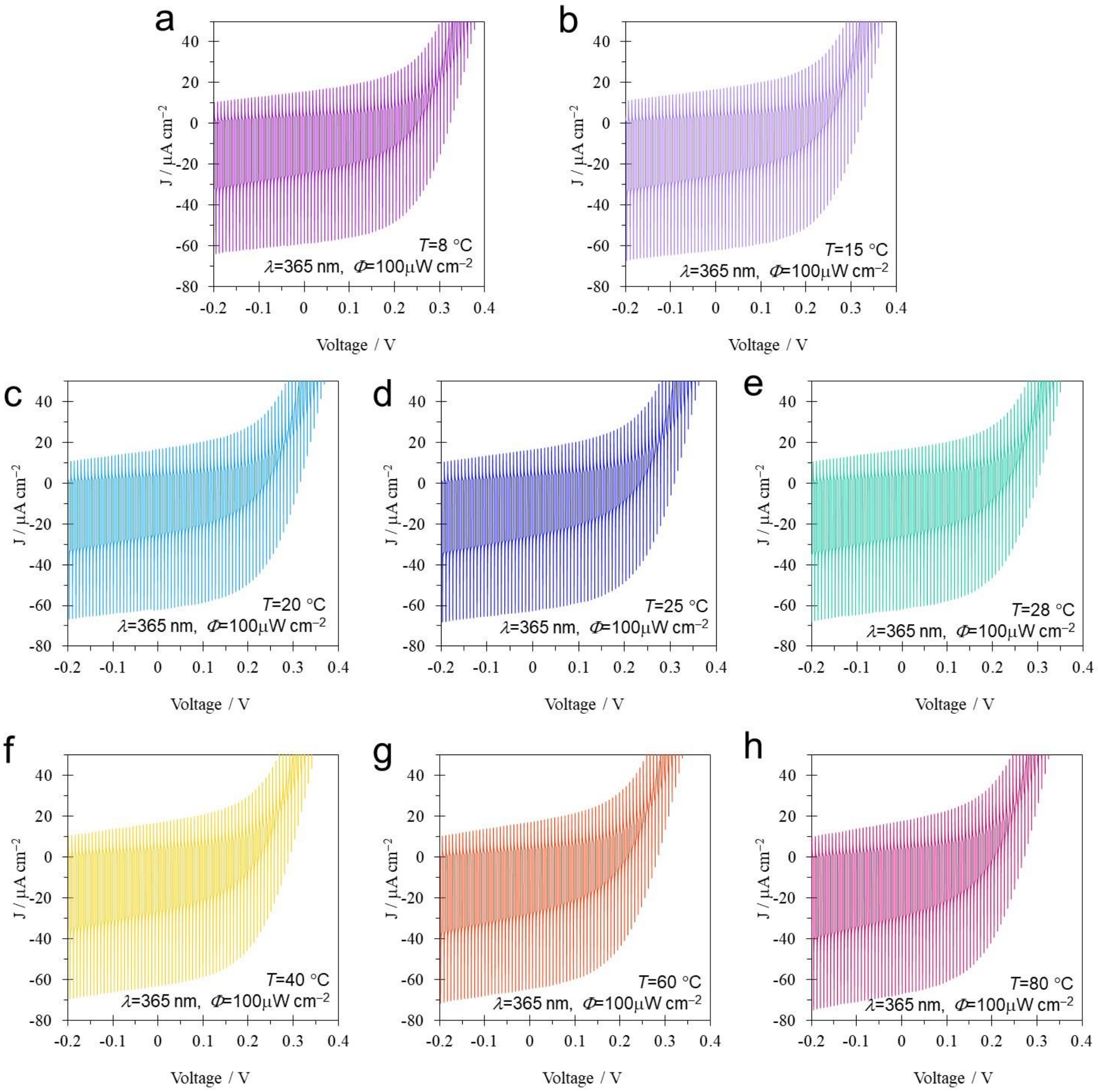

Supplementary Fig. 28 | Current-voltage characteristics of the TPV device under various temperatures (T). (a) T = 8 °C. (b) (a) T = 15 °C. (c) T = 20 °C. (d) T = 25 °C. (e) T = 28 °C. (f) T = 40°C. (g) T = 60 °C. (h) T = 80 °C. Throughout these measurements, scan speed and sample interval were 0.5 V s^{-1}, and 5 μV, respectively. Light illumination wavelength, intensity, and pulsed frequency were 365 nm, 100 μW cm^{-2} and 60 Hz, respectively.

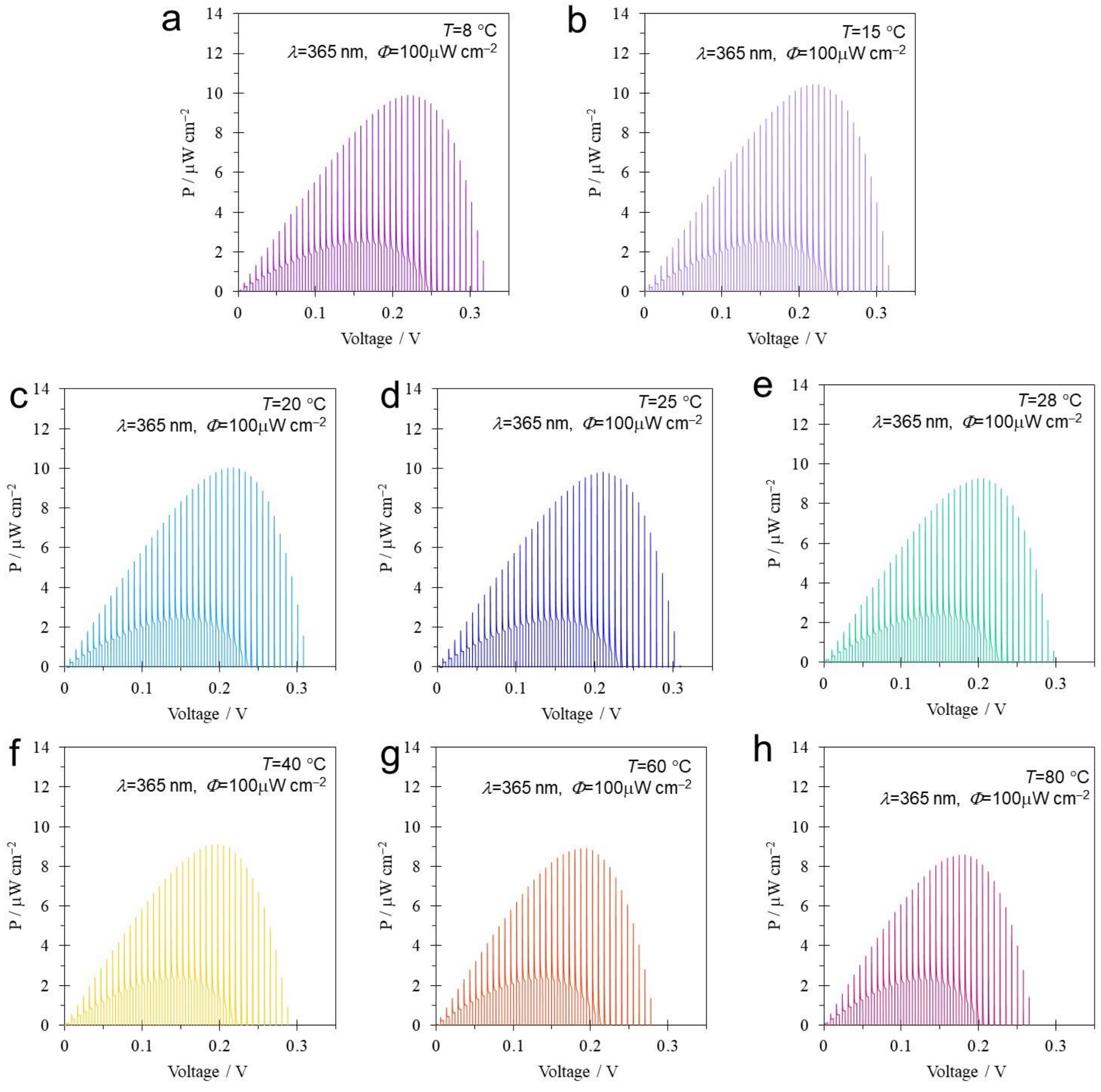

Supplementary Fig. 29 | Power-voltage characteristics of the TPV device under various temperatures (T). (a) T = 8 °C. (b) (a) T = 15 °C. (c) T = 20 °C. (d) T = 25 °C. (e) T = 28 °C. (f) T = 40°C. (g) T = 60 °C. (h) T = 80 °C. Throughout these measurements, scan speed and sample interval were 0.5 V s^{-1}, and 5 μV, respectively. Light illumination wavelength, intensity, and pulsed frequency were 365 nm, 100 μW cm^{-2} and 60 Hz, respectively.

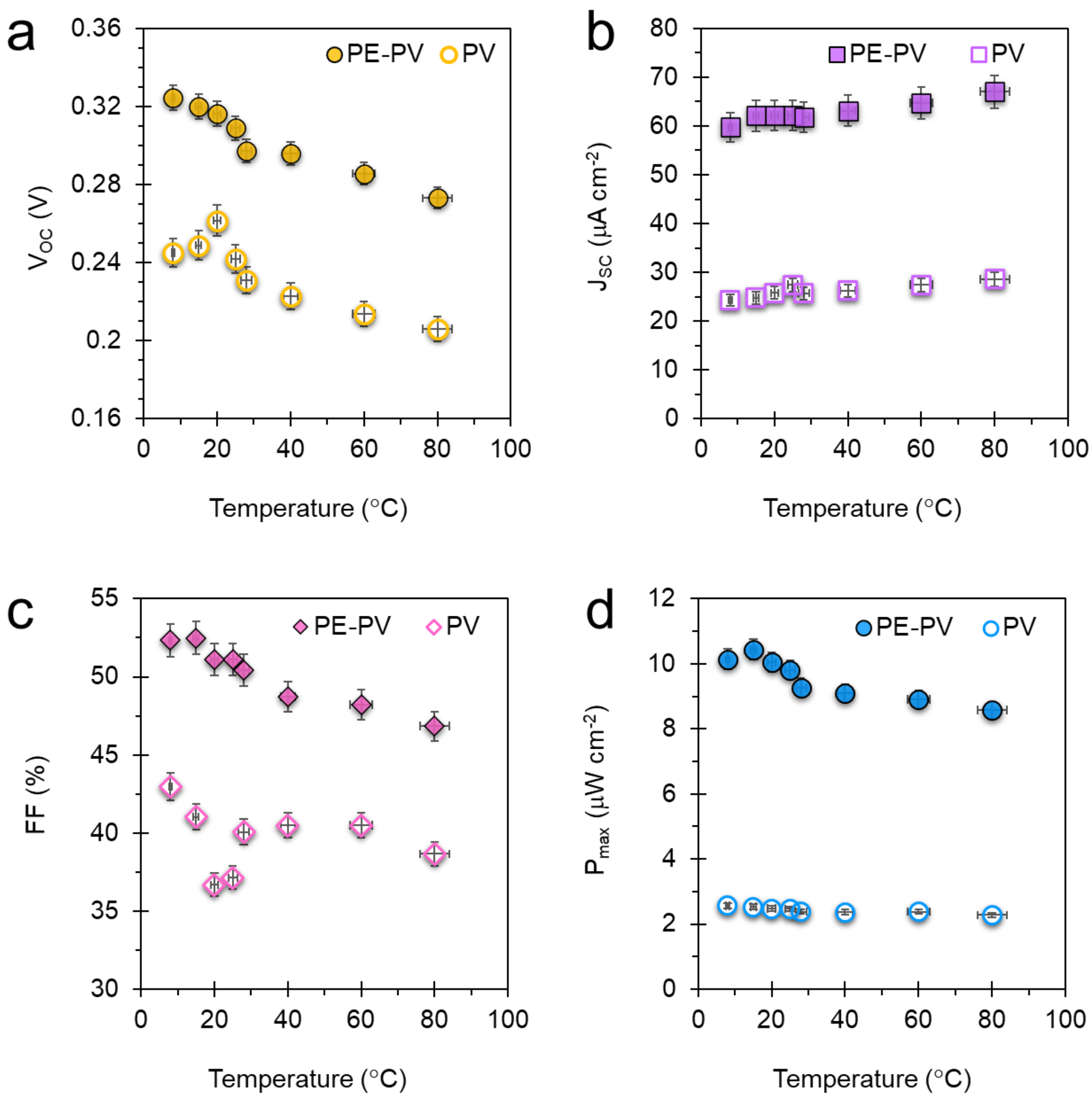

Supplementary Fig. 30 | Summary of the performance parameters of the pyro-TPV devices as a function of device temperature. (a) V_{OC}, (b) J_{SC}, (c) FF, and (d) P_{max} versus temperature (error bar is 5%). These parameters were obtained from the J-V characteristic and P-V plots in Supplementary Figs. 28 and 29.

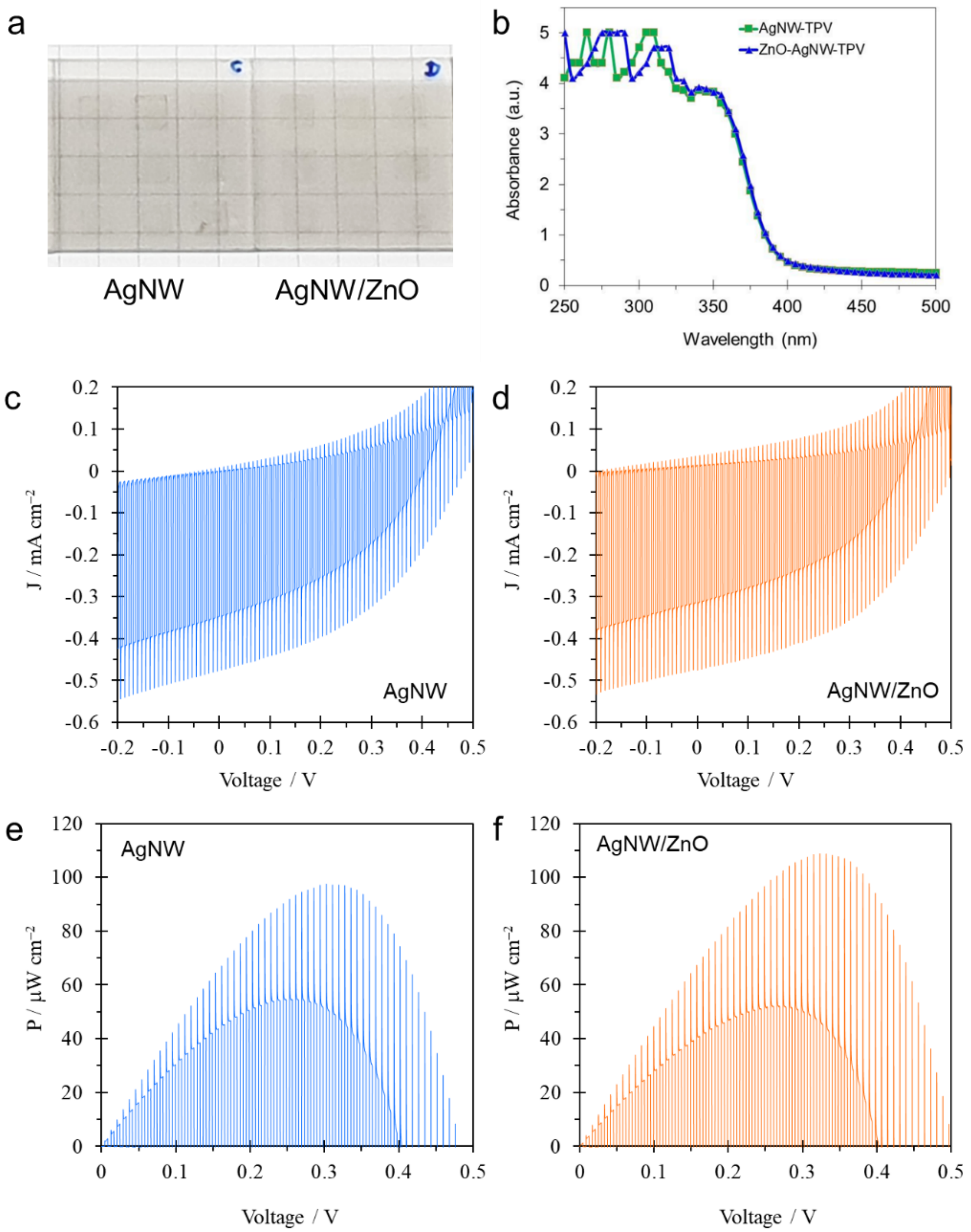

Supplementary Fig. 31 | (a) Transparent pyroelectric heterojunction device array with AgNW and AgNW/ZnO electrode. (b) Absorbance spectra of the devices with AgNW and AgNW/ZnO electrodes. Current density-voltage (J-V) characteristic plot of the TPHD with (c) AgNW and (d) AgNW/ZnO electrode. Power density-voltage (P-V) characteristic plot of the TPHD with (e) AgNW and (f) AgNW/ZnO electrode. These array devices were measured under the pulsed illumination wavelength of 365 nm, an intensity of 1.4 mW cm^{-2}, a pulse frequency of 60 Hz with a duty cycle of 50%, a scan speed of 0.5 V s^{-1}, and a sample interval of 10 μV. The illumination direction was from the FTO side, while the Au pogo pin was applied from the top electrode to avoid possible parasitic absorption and artifacts from non-uniform illumination.

Supplementary Table 3 | Summary of the performance parameters of the device with AgNW and AgNW/ZnO electrode

Parameters	AgNW		AgNW/ZnO	
	PV	PE-PV	PV	PE-PV
J_{SC} (mA cm^{-2})	0.347	0.47776	0.314	0.4754
V_{OC} (V)	0.404	0.4847	0.401	0.4982
P_{max} (μW cm^{-2})	54.87	97.54	52.00	108.8
IPCE (%)	84.28	115.93	76.19	115.35
FF (%)	39.16	42.12	41.43	45.93
PCE (%)	3.92	6.97	3.73	7.77

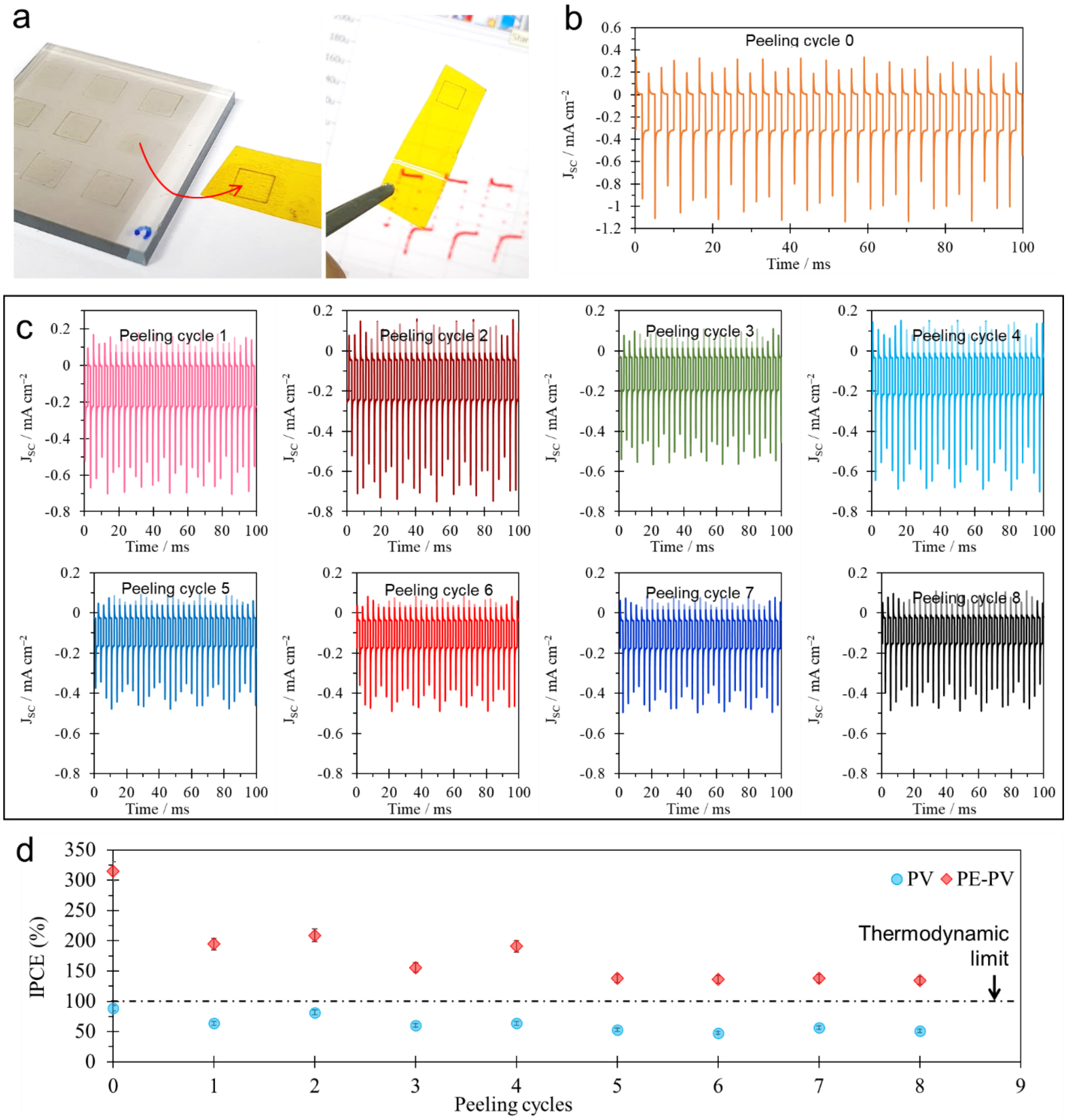

Supplementary Fig. 32 | Adhesion test of the device with AgNW/ZnO electrode. (a) The original photo image of the device after the adhesion test by the Kapton tape. (b) The short-circuit current density (J_{SC}) profile under the pulse illumination wavelength of 365 nm and intensity of 1.2 mW cm^{-2} before the adhesion test. (c) The J_{SC} profiles of the device after Kapton tape peeling cycles from 1 to 8. (d) Incident photon to current conversion efficiency (IPCE) of the device for the PV and PE-PV phenomena are presented as a function of peeling cycles (error bar is 5%). It is worth noting that the thermodynamic limit was set at 100% of the IPCE value.
